# RNase H-dependent PCR enables highly specific amplification of antibody variable domains from single B-cells

**DOI:** 10.1371/journal.pone.0241803

**Published:** 2020-11-05

**Authors:** John Crissman, Yuhao Lin, Kevin Separa, Madeleine Duquette, Michael Cohen, Candyd Velasquez, Thomas Cujec

**Affiliations:** 1 Department of Biologics Automation and High-Throughput Technologies, Eli Lilly and Company Biotechnology Center, San Diego, California, United States of America; 2 Department of Biologics IT, Eli Lilly and Company Biotechnology Center, San Diego, California, United States of America; University of Helsinki, FINLAND

## Abstract

Immunization-based antibody discovery platforms require robust and effective protocols for the amplification, cloning, expression, and screening of antibodies from large numbers of B-cells in order to effectively capture the diversity of an experienced Ig-repertoire. Multiplex PCR using a series of forward and reverse primers designed to recover antibodies from a range of different germline sequences is challenging because primer design requires the recovery of full length antibody sequences, low starting template concentrations, and the need for all the primers to function under the same PCR conditions. Here we demonstrate several advantages to incorporating RNase H2-dependent PCR (rh-PCR) into a high-throughput, antibody-discovery platform. Firstly, rh-PCR eliminated primer dimer synthesis to below detectable levels, thereby eliminating clones with a false positive antibody titer. Secondly, by increasing the specificity of PCR, the rh-PCR primers increased the recovery of cognate antibody variable regions from single B-cells, as well as downstream recombinant antibody titers. Finally, we demonstrate that rh-PCR primers provide a more homogeneous sample pool and greater sequence quality in a Next Generation Sequencing-based approach to obtaining DNA sequence information from large numbers of cloned antibody cognate pairs. Furthermore, the higher specificity of the rh-PCR primers allowed for a better match between native antibody germline sequences and the VL/VH fragments amplified from single B-cells.

## Introduction

In 2018, there were 64 FDA-approved therapeutic antibodies on the market with total sales exceeding $100 billion. As additional antibody-based therapeutics come to market, global sales are projected to reach $300 billion by 2025 [1–3]. Undoubtedly, productive antibody discovery platforms will continue to play a critical role towards delivering on this important class of therapeutics.

Numerous criteria must be considered when developing a therapeutic antibody to a target, e.g., binding affinity, appropriate epitope to elicit the desired functional response, target selectivity, high expression levels, low immunogenicity risks, low non-specific binding, and minimal developability liabilities. Notably, these three latter criteria can affect pharmacokinetic and disposition properties. It is because of these exacting criteria that large numbers of antibodies are typically cloned, expressed, and evaluated as part of a discovery campaign to identify optimal hits. Numerous antibody discovery platforms are currently in wide-spread use including immunization-based approaches in transgenic or non-genetically modified animals, *in vitro* and *in vivo* display platforms, as well as retrieval of human B-cells from infected patients [4–6].

As part of the adaptive immune response in vertebrates, the Variable (V), Diversity (D), and Joining (J) gene segments, referred to as V(D)J, in B-cells recombine to form the variable heavy chain (VH) of an antibody, while the V and J gene segments (V-J) on a different chromosome recombine to form the variable light chain (VL). After recombination of the VL and VH germline sequences, the B-cells leave the bone marrow and enter circulation and secondary lymphoid tissues. A specific recognition event between the B-cell receptor expressed on the cell surface and a foreign protein (antigen), results in proliferation of the B-cell, affinity maturation of the antibody through a process of somatic hypermutation and isotype class-switching of the heavy chain constant domain from an IgD or IgM to IgA, IgE, or IgG [7, 8]. The immune repertoire of vertebrates commonly used in discovery platforms contains between 40–60 distinct germline VH sequences or isotypes and 4–6 J-region sequences [9]. Since the antibody sequences expressed by B-cells are not known *a priori*, a series of forward and reverse primers are required to amplify the VL and VH genes from larger numbers of B-cells to capture the diversity of the immune response following immunization.

We and others have developed protocols for the amplification, cloning and expression of antigen-specific antibodies from single B-cells isolated from immunized animals or human plasma cells [10–13]. The need to recover full-length VL and VH domains from B-cells limits the endogenous sequences that can be used as anchor points for PCR primers. Typically the forward PCR primers are designed to hybridize to the signal (leader) sequences responsible for secretion of the antibody from the cell, or to Framework 1 (FR1) sequences at the 5’ terminus of the variable region, while the reverse primers hybridize to either the J-regions of the antibody variable regions, or the constant sequences (IgG, kappa, lambda). All the primers must function under the same multiplex PCR conditions. Cognate pairing between the VL and VH chains recovered from a single B-cell is maintained by their co-ordinate well positions in a microtiter plate. The original single cell PCR (scPCR) methods have been adapted and streamlined to enable high-throughput cloning and expression of antibodies from large numbers of B-cells to maximize the capture of antibody diversity across the immune repertoire.

RNase H2-dependent PCR (rh-PCR) utilizes DNA primers that contain a single ribonucleotide base upstream of a 3’ blocking group [14]. Participation of the primers in the PCR amplification process is dependent upon cleavage of the 3’ blocking group by the *Pyrococcus abyssi* RNase H2 enzyme. Cleavage of the phosphodiester bond 5’ of the ribonucleotide by the RNase H2 enzyme is required for participation of the primer in the amplification reaction and occurs only upon primer-template duplex formation. Furthermore, efficient cleavage by RNase H2 is dependent on correct base pairing between the template and the primer in the vicinity of the ribonucleotide. Since the enzyme is thermostable (50°C–75°C), and has minimal activity at room temperature, it can be added directly to the PCR mix and confers a stringent “hot-start” functionality to the PCR.

RNase H-dependent PCR primers can be used in end-point PCR as well as qPCR. The use of rh-PCR primers in an amplification reaction has been demonstrated to increase the specificity of amplification, improve reproducible recovery of low abundance targets, and decrease the formation of primer dimers. RNase H-dependent PCR has been used to recover paired alpha/beta T-cell receptor pairs from single cells, as well as T-cell receptor analysis of bulk RNA samples [15]. In addition, rh-PCR has been used to detect mRNA splice mutants [16], as well as genotyping assays to detect single-nucleotide polymorphisms in human, plant samples, and microorganisms [17–19].

Typically, antibodies are initially selected for further study based on their binding affinities, species cross-reactivity, epitope diversity, and functional activity in cell assays [20, 21]. However, the developability and immunogenic properties of an antibody are also important for ease of manufacturing, formulation, storage, and to ensure adequate dosing, drug disposition, and *in vivo* efficacy. A wide variety of analytical assays have been developed for assessment of important developability factors such as the molecular heterogeneity of the molecule, chemical stability, thermal stability, propensity for self-association, and solubility [22]. In addition, *in silico* modeling of antibodies, based on their amino acid sequence, are being developed to predict post-translational modifications, aggregation, viscosity, hydrophobicity, electrostatic properties, and immunogenicity. These *in silico* predictions are being validated using empirical data obtained from high-throughput analytical experiments and evaluated in conjunction with data from manufacturing and animal studies to predict biophysical and drug properties [23]. The goal is to develop machine-based learning algorithms that will allow assessment of 10,000s of antibodies and reduce the amount of hands-on experimentation required in the laboratory. Development of robust *in silico* antibody assessment tools is predicated on obtaining many DNA sequences for a diverse set of antibodies in a facile and cost-effective manner.

Next Generation Sequencing (NGS) has played an increasingly important role in the discovery and engineering of novel antibodies. High-throughput sequencing of bulk B-cells from immunized animals, and humans infected with bacterial or viral pathogens has been used to understand B-cell development and antibody repertoire diversity, as well as for the identification of target-specific antibodies and their affinity-matured daughters [24–27]. More recently, microfluidic emulsion-based approaches have been developed to maintain the cognate pairing between the VL and VH antibody chains from single B-cells thereby greatly improving the affinity and specificity of recovered antibodies [28, 29]. NGS has also been used to identify target-specific binders from antibody libraries displayed on phage, bacteria or yeast, as well as from a variety of *in vitro* display formats [26, 30–32].

In protein engineering applications, NGS has been used to interrogate enriched pools following panning of phage or yeast display libraries, as well as *in vitro* display systems from libraries containing large numbers of mutants. Deep mutational scanning has been used to identify mutagenized proteins with improved affinity and specificity to a desired target, enzymes with increased catalytic activity, to inform protein structure analysis, as well as for the identification of thermodynamically stabilizing mutations [33, 34].

This research demonstrates the applicability of rh-PCR in the discovery of novel antibodies. The results show that rh-PCR reduces primer dimer amplification to undetectable amounts resulting in accurate quantification of antibody titers and the elimination of wells lacking a full-length antibody sequences (false IgG+) from downstream screening. The greater specificity and robustness of amplification reactions containing rh-PCR primers results in a larger number of antigen-specific binders recovered from single B-cells, and higher antibody titers following recombinant expression in mammalian cells. Lastly, the work demonstrates that the increased specificity of rh-PCR primers relative to standard primers improves the quality and number of Next Generation Sequencing reads from cloned samples, as well as a closer nucleotide match between native antibody germline sequences and the VL/VH fragments amplified from single B-cells.

## Material and methods

### PCR amplification of variable regions

Following mouse immunizations, single antigen positive B-cells were FACS sorted into 96-well PCR plates (Bio-Rad) containing 10 μL of Lysis buffer [1.25X First strand buffer (Thermo Fisher), 6.25 mM DTT (Thermo Fisher), 0.3% NP40 (Thermo Fisher), 5 U RNaseOUT (Thermo Fisher), 0.02 μg/μL tRNA (Sigma)], and the plates stored at -80°C. Prior to processing, the plates were thawed, cDNA synthesis performed by adding 3 μL Master mix [25 U SuperScript III reverse transcriptase (Thermo Fisher), 225 ng random hexamers (Gene Link), 3.3 mM dNTP mix (Qiagen)] to each well and reacted on a Bio-Rad C-1000 thermocycler (42°C, 10 min; 25°C, 10 min; 50°C, 60 min; 94°C, 5 min). The cDNA served as the template for amplification of the variable regions with a pool of primers complimentary to the germline leaders and constant regions. cDNA, 2 μL, was transferred to 10 μL master mix [1X Phusion HF buffer (NEB), 0.24 mM dNTP mix (Qiagen), 0.5 μM primers (IDT), 0.2 U Phusion high-fidelity DNA polymerase (Bio-Rad)] and amplified on a Bio-Rad C-1000 thermocycler (98°C, 30 sec; (98°C, 1 sec 68°C, 30 sec; 72°C, 60 sec) x 50 cycles; 72°C, 10 min). To prepare the variable regions for cloning, the products of the previous PCR were amplified using a pool of primers complimentary to the antibody FW-1 and the J-regions. Using a 1:100 dilution of the previous PCR reaction, 1 μL was transferred to 10 μL master mix [1X Phusion HF buffer (NEB), 0.24 mM dNTP mix (Qiagen), 0.5 μM primers (IDT), 0.2 U Phusion high fidelity DNA polymerase (Bio-Rad)] and amplified on a Bio-Rad C-1000 thermocycler (98°C, 30sec; (98°C, 10 sec; 65°C, 30 sec; 72°C, 60 sec) x 34 cycles; 72°C, 10 min). This second PCR was done using a standard primer set as well as primers modified for both the rh-PCR generation 1 and generation 2 primer designs (Integrated DNA Technologies, IDT). For amplifications involving rh-PCR primers, the reactions were supplemented with 40 mU RNase H2 enzyme (IDT), prepared by a 50-fold dilution of the stock into Dilution Buffer (IDT). All primer sets contained 5’ bases complimentary to the expression vector to allow for cloning by the Gibson method.

### Agarose gel electrophoresis

The 96-well electrophoretic separation of the second PCR products were done by loading 1.5 μL onto E-gel 96–2% Agarose gels (Thermo Fisher) using a Beckman Biomek-FX liquid handler followed by electrophoresis for 5 minutes. Visualization and documentation of the gels was performed on an AlphaImager HP (Protein Simple) equipped with a UV transilluminator box (260nm). DNA samples were also electrophoresed on 1.2% FlashGels (Lonza) and similarly documented.

### Capillary electrophoresis

PCR products were separated by capillary electrophoresis using the Infinity 96-well instrument (Agilent) running the DNF-935 dsDNA Reagent Kit (1–1500 bp). PCR samples were diluted 1:12 in sample Dilution Buffer in 96-well PCR plates prior to loading onto the instrument and electrophoresed in parallel. Analysis and overlays of the resulting electropherograms were done using the PROSize 3.0 software (Agilent).

### Primer-Phusion time course

The VH forward Generation 1 rh-PCR primer pool was diluted to a concentration of 0.8 μM in 125 μL of either 1X Phusion Hi-Fi Buffer (NEB) or 1X Exonuclease I Buffer (NEB). Aliquots of these reactions, 10 μL, were dispensed into replicate wells of three 96-well PCR plates and additions of either 1 μL (2U/μL) Phusion High Fidelity DNA Polymerase (NEB), 1 μL (20U/μL) Exonuclease I (NEB), or 1 μL water were made at T_0_. The plates were incubated at 37°C and at timepoints of 1 hour, 3 hours, and 6 hours one plate was removed from incubation and 2 μL of the reactions were analyzed by capillary electrophoresis as described above.

### Exonuclease digestion of PCR products

To digest the unincorporated primer pools in the completed PCR reactions prior to capillary electrophoresis, 5 μL of PCR product was transferred to wells of a 96-well PCR plate and 1 μL of either Exonuclease I (20U/μL)(NEB) or Exonuclease VII (10U/μL)(NEB) were added. The plate was incubated at 37°C for 30 minutes followed by a heat inactivation step of 95°C for 10 minutes. The samples were then analyzed by capillary electrophoresis as described above.

### Cloning and expression of antibody variable region fragments

Variable regions from the second PCR amplification were diluted 1:30 in water and 1 μL mixed with 20 ng of linearized expression vector in 4 μL reactions containing Gibson Assembly Master Mix (NEB). The reactions were incubated at 50°C for 60 minutes. From the Gibson reactions, 1 μL was used to transform competent *E*. *coli* to Carbenicillin resistance and subsequently to inoculate 1 mL 2X YT medium (Thermo Fisher) supplemented with 100 μg/mL Carbenicillin in 96 deep well blocks. After 20 hours of growth in a shaking incubator at 37°C, the cells were harvested by centrifugation and the plasmids isolated using the Plasmid Plus 96 Kit (Qiagen). Plasmid DNA concentrations were determined spectrophotometrically using a Spectramax 384 Plus spectrophotometer. Plasmids containing the variable light plus kappa constant and variable heavy plus IgG4 constant regions, and originating from the same B-cell, were mixed using the Lynx LM I800 VVP (Dynamic Devices) into 96 deep well Master-blocks (Greiner) and used to transiently transfect CHO cells using Polyethyleneimine (PEI) as described previously [35]. On day six post-transfection, the supernatants containing the expressed antibodies were harvested.

### Quantification of IgG

The CHO supernatants were diluted 1:5 in PBS (Corning) and 40 μL each sample transferred to the wells of a Tilted-bottom (TW384) Microplate (forte’BIO). The test samples were measured using Protein G (ProG) Dip and Read Biosensors (forte’BIO) on a high-throughput Octet Red 384 system (forte’BIO). Sample values were fit to a stored human IgG4 standard curve and final antibody concentrations calculated using the Data Analysis HT (ver. 10) software.

### SDS-PAGE analysis of expressed antibody supernatants

To visualize the size of the protein expressed by the in-frame cloning of small PCR fragments, CHO supernatants were diluted in 4X PAGE sample buffer (Thermo Fisher) both with and without β-ME (Thermo Fisher). After heating the samples to 100°C for 5 minutes, 50 ng of IgG (by Octet) were loaded onto a 4–12% BIS-TRIS PAGE (Thermo Fisher) and electrophoresed at 100V for 90 minutes. The separated proteins were transferred to nitrocellulose for 7 minutes on the iBlot Western Blotting System (Thermo Fisher), blocked for 1 hour in Casein Blocker (Thermo Fisher), and then incubated overnight in a 1:2000 dilution of Goat anti-Human IgG-Fc- AP (Southern Biotech) in Casein Blocker. Following three 10-minute washes in TBS + 0.1% Tween-20, the blot was developed using 1-Step NPT/BCIP (Thermo Fisher) substrate and visualized on an AlphaImager HP (Protein Simple).

### Antigen-down ELISA

Soluble antigen was diluted to 2 μg/mL in Carbonate Coating Buffer (0.2 M Carbonate-Bicarbonate, pH 9.4 (Thermo Fisher)) and 20 μL was dispensed into the wells of a 384-well High Binding microplate (Greiner). The plate was sealed and incubated at 4°C overnight. After washing 3X with PBS + 0.1% Tween-20 (in house), the plate was blocked with 80 μL of Casein Blocker in PBS (Thermo Fisher) for 60 minutes. Following washing, 20 μL of normalized CHO supernatants at 5, 1.25, 0.31 and 0.08 μg/mL were added to the wells of the ELISA plate and incubated for 60 minutes. The expression supernatants derived from minipreps for both the standard primers and the rh-PCR Generation 2 primers were tested. After washing 3X with PBS + 0.1% Tween-20, 20 μL of 1:1000 dilution of Goat anti-Human kappa-AP (Southern Biotech) in Casein Blocker was added to each well and incubated 60 minutes. After washing again, 20 μL of 1-Step PNPP (Thermo Fisher) was added and the plate incubated until color developed. The plate was read on a Spectramax 384 Plus spectrophotometer at 405 nm.

### Preparation of samples for NGS

Using an Echo 525 Acoustic Liquid Handler (Beckman), 1.6 ng of miniprep DNA was transferred into 384-well PCR plates containing 10 μL Master mix [1X Phusion HF buffer (NEB), 0.24 mM dNTP mix (Qiagen), 0.5 μM primers (IDT), 0.2 U Phusion high fidelity DNA polymerase (Bio-Rad)] and amplified on a Bio-Rad C-1000 thermocycler (98°C, 30 sec; (98°C, 10 sec; 55°C, 30 sec; 72°C, 60 sec) x 20 cycles; 72°C, 10 min’). Upon completion the PCR products were purified using MagBind RXP magnetic beads (Omega Bio-tek) at 0.6x ratio and eluted in 20 μL 10 mM Tris pH 8. Unique NGS index sequences were added to the variable region fragments in a second PCR amplification, 2.5 μL of the bead purified first PCR product was transferred into the wells of a 384-well PCR plate containing 5 μL of 2X KAPA Premixed Master mix (Roche) and 2.5 μL 1 μM Index primers (IDT), and amplified on a Bio-Rad C-1000 thermocycler (95°C, 3 min; (95°C, 30 sec; 55°C, 30 sec; 72°C, 30 sec) x 8 cycles; 72°C, 5 min). The amplified products were pooled, purified using MagBind RXP magnetic beads (Omega Bio-tek) (0.6x) and eluted from the magnetic beads in 200 μL TE. The library was quantified by qPCR (KAPA Library Quantification Kit) on a ViiA 7 Real-Time PCR System (Applied Biosystems). An Agilent DNA 1000 Kit was used with a 2100 Bioanalyzer (Agilent) for quality control of the library. The library was diluted to 4 nM in 10 mM Tris HCl pH 8.0 and denatured according to the “MiSeq System Denature and Dilute Libraries Guide” to 8 pM with 20% PhiX spiked into the solution. The denatured library was sequenced on a MiSeq (Illumina) and used a MiSeq Reagent Kit v2 (15M reads). The MiSeq read 250 x 250 cycles and generated 16.57 M raw reads with 89.55% reads passing filter at 89.30% > = Q30.

### Analysis of next generation sequencing results

We applied our in-house single-cell NGS analysis pipeline on the same samples amplified, separately, from both standard primers and rh-PCR primers. First, the outer forward and reverse primers targeting the leader and constant regions were removed from the raw reads using Cutadapt 1.9.1 [36]. The paired-end reads were then stitched to reconstitute the original VH/VL sequences using Flash2 [37], based on the overlap between R1 and R2. ANARCI 1.3 [38], which aligns potential antibody sequences to Hidden Markov Models, was used to identify productive antibody sequences and their closest V- and J-genes in each well. To analyze the prevalence of full length sequences in each well, we considered VH sequences ≥ 111 residues and VL sequences ≥ 106 residues to be full length for the purpose of this analysis; the minimum sequence lengths are the minimum combined V and J gene length from the IMGT human germline reference sequences included in ANARCI 1.3, for VH and VL respectively. To evaluate the specificity of standard versus rh-PCR primers, we considered all sequences in each well from the same clonotype (defined as having identical CDR3 sequence under the North CDR definition [39] as the most abundant antibody sequence in that well; we calculated the percentage of those sequences that matched perfectly the primer-annealing region (first 21 nucleotides of FR1) of the V-gene of that clonotype.

## Results

### Transfections with non-functional VH domains results in aberrant IgG titers

We have developed a high-throughput procedure for amplifying cognate VL/VH pairs from single, target-specific B-cells following immunization of a variety of animal species. Following cDNA synthesis, a separate amplification reaction (PCR #1) is done using primers that anneal to the signal peptide sequences upstream of the antibodies variable light (VL) or heavy VH (VH) sequences and the constant kappa/lambda or IgG domains. A nested PCR (PCR #2) is then performed using primers that anneal to the framework 1 (FR1) and J-region sequences of the antibody variable regions. These primers contain 5’ sequences that overlap the signal peptide and IgG constant regions of our mammalian expression vectors, thereby allowing for cloning by Gibson assembly. After cloning, DNA is extracted from the bulk *E*. *coli* transformants and used to transfect CHO cells in a 96-well plate format. Subsequent antibody titers are determined based on an intact IgG-Fc domain using Octet biosensor probes and tested via ELISA for binding to the antigen used in the animal immunizations.

**Fig 1:**
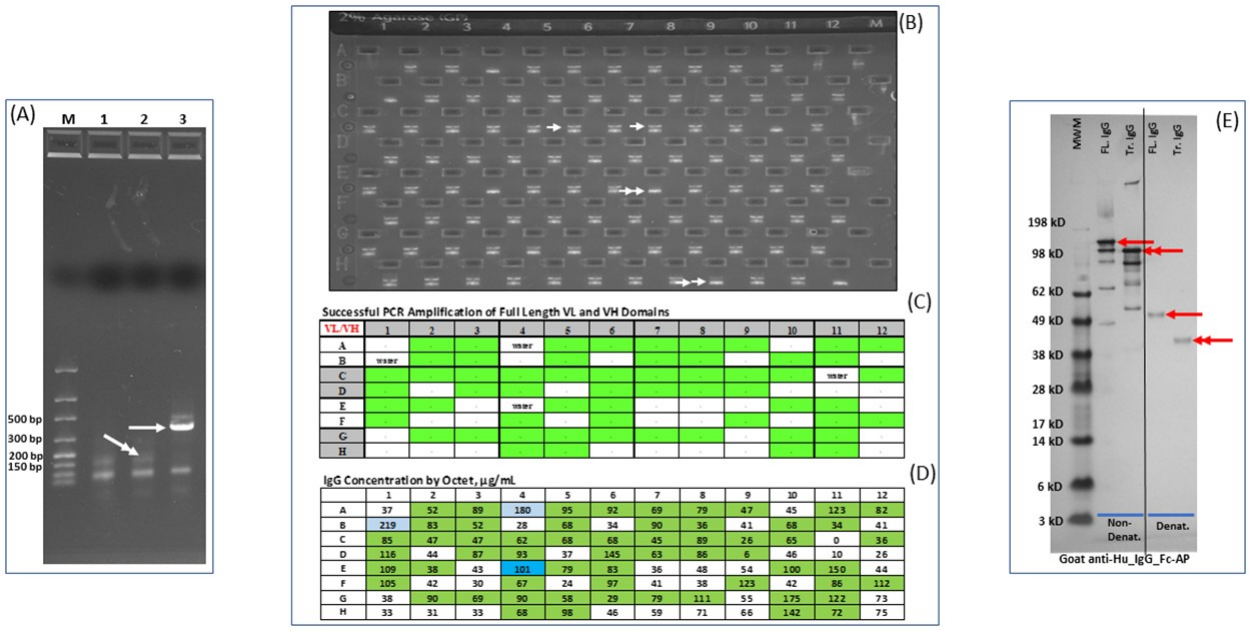
PCR amplification of VH fragments from single B-cells. (A) Amplification of VH fragments using standard FR1 and J-region primers results in full-length product (arrowhead), as well as a truncated fragment of <150 bp in size (double arrowhead). (B) A representative gel of a series of VH fragments. (C) Recovery of cognate, full-length VL and VH antibody domains from single B-cells using framework 1 and J-region primers. The green box denoted recovery of cognate full length VL and VH fragments. Wells not receiving a B-cell are designated as water wells. (D) Ab titers following transient expression of cognate VL and VH pairs in CHO cells. Light and dark blue cells denote transfection and Octet controls, respectively. (E) Western blot analysis of antibodies containing either a full-length or a truncated VH fragment. Antibodies containing full-length or truncated VH fragments are indicated with a single or double arrowhead respectively.

As part of the QC process, products from the nested PCR were separated on an agarose gel, and wells scored for the presence of either a VL or a VH band of the expected size. A representative agarose gel of a series of VH fragments is shown (Fig 1A and 1B). In addition to the expected full-length VH product (350–400 bp), a small PCR product of < 150 bp was also apparent (Fig 1A and 1B).

As part of data tracking and process optimization, successful recovery of cognate VL and VH chains from a single B-cell following PCR is determined and denoted by a green box (Gr/Gr pair) (Fig 1C). In this experiment, both VL and VH fragments of the expected size were detected in 59 of the 92 wells (64%) having single B-cells, while the remaining 33 wells were missing a band corresponding to either the VL domain, the VH domain, or both. The DNA from cognate VL and VH pairs were combined in a 1:1 ratio in a 96-deep well block and expressed in CHO cells. As expected, control antibody titers (wells A4 and B1) averaged around 200 μg/mL (Fig 1D). The average antibody titers for test samples containing plasmids with full-length VL and VH fragments was 80 μg/ml, while the average titers for wells containing plasmids missing at least one of the variable regions was 43 μg/mL. Significantly, in every instance (33 wells) where at least one of the antibody variable domains was missing, titers were still above our threshold for calling an expressed sample (>5 μg/mL). Western blot analysis using a detection antibody specific against the human IgG-Fc domain demonstrated that the small PCR fragments encoded a truncated heavy chain with a functional Fc domain (Fig 1E), suggesting that these molecules can likely bind to the Octet bio-sensor and result in a false positive antibody titer reading.

A sampling of cloning reactions from wells containing full-length VH domains, as well as those containing only truncated PCR products were transformed into *E*. *coli* and single colonies analyzed by Sanger sequencing. Data from four discovery projects are summarized in Table 1. Of the 156 colonies transformed with cloning reactions containing full-length VH fragments, 150 clones (96.2%) contained full-length sequences. The other six colonies had full-length VH domains that were out of frame from the upstream leader sequence. In all wells containing a full-length VH fragment, Sanger sequencing of single colonies failed to identify a single case of the smaller fragment being cloned. This suggests that cloning of the full-length VH domains into the mammalian expression vectors is efficient and that plasmid DNA isolated from bulk *E*. *coli* transformants will contain a high proportion of productive Gibson assembly reactions, resulting in high antibody titers. All 59 colonies transformed with truncated PCR products contained exclusively small fragments (<150 bp).

**Table 1:** Single colony sequence analysis of VH amplicons following cloning into mammalian expression vectors.

			Sequencing of Individual Clones		
Project	Plate_Well	VH-PCR Band	# Readable Sequences	Full Length, in frame VH	<50 bp in frame product
**1**	**18_A3**	**+**	**8**	**8**	
	**18_A5**	**+**	**8**	**8**	
	**18_B2**	**+**	**8**	**8**	
	**18_B3**	**+**	**8**	**8**	
	**18_B7**	**+**	**8**	**8**	
	**18_A7**	**-**	**8**		**8**
	**18_A11**	**-**	**8**		**8**
	**18_F12**	**-**	**8**		**8**
**2**	**1_F4**	**+**	**8**	**8**	
	**1_A10**	**+**	**8**	**8**	
	**2_C1**	**+**	**8**	**8**	
	**2_D10**	**+**	**8**	**8**	
	**1_H3**	**-**	**8**		**8**
	**2_C12**	**-**	**8**		**8**
**3**	**2_3H**	**+**	**7**	**7**	
	**2_A7**	**+**	**7**	**7**	
	**3_E1**	**+**	**6**	**6**	
	**3_D10**	**+**	**8**	**8**	
	**3_B12**	**+**	**8**	**2**^1^	
	**24_A1**	**+**	**8**	**8**	
	**24_E9**	**+**	**8**	**8**	
	**2_B6**	**-**	**8**		**8**
	**24_H9**	**-**	**8**		**8**
**4**	**4_A10**	**+**	**8**	**8**	
	**4_B10**	**+**	**8**	**8**	
	**4_C10**	**+**	**8**	**8**	
	**4_D10**	**+**	**8**	**8**	
	**4_G9**	**-**	**1**		
	**4_C11**	**-**	**1**		**1**
	**4_A11**	**-**	**1**		**1**

^1^ 6 of 8 clones Full Length but Out of Frame.

Sequencing of single colonies from cloning reactions containing the truncated PCR fragments demonstrated that the truncated PCR product was comprised of a short sequence flanked by the forward (FR1) and reverse (J-region) primers used in the nested PCR. This fragment was in-frame with the signal peptide and CH1 sequences of the mammalian expression vector (S1 Fig). Despite extensive efforts, the presence of the truncated PCR product was essentially unaffected by adjustments in primer annealing temperatures, reduction in primer concentrations, adjustments in the forward to reverse primer ratio, or a reduction in PCR cycles.

### Characterization of rh-PCR primer designs

**Fig 2:**
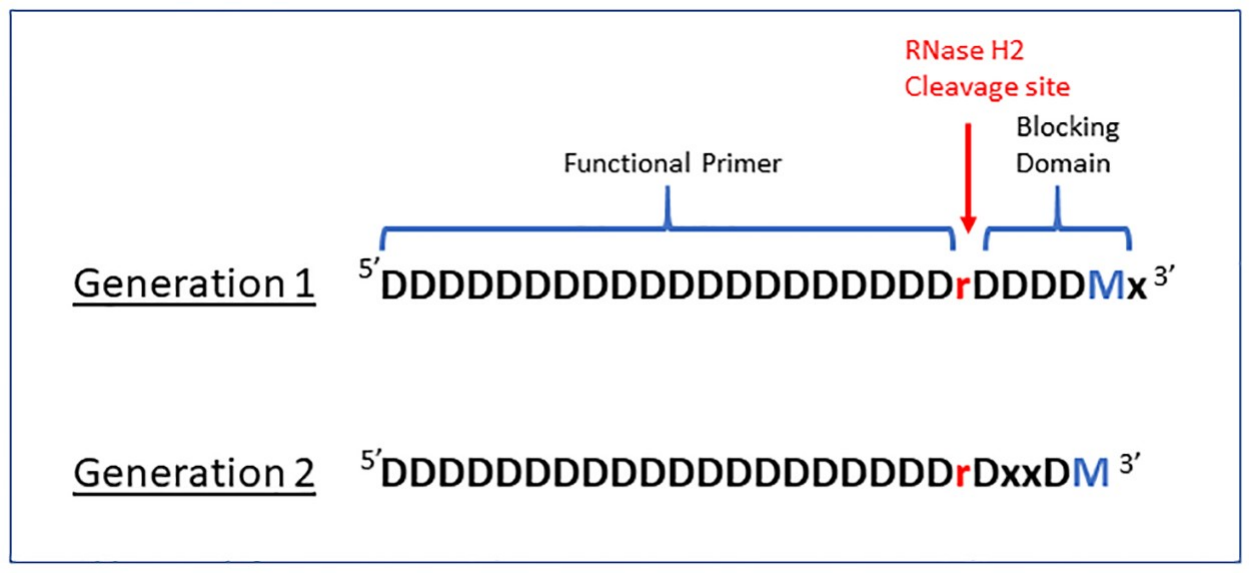
IDT Blocked cleavable primers. Design comparison of IDT’s Generation 1 versus Generation 2 Blocked Cleavable Primers for use in rh-PCR. After annealing to the target sequence, the thermostable RNase H2 enzyme cleaves at the single ribonucleotide releasing the Blocking Domain allowing for primer extension. The Generation 2 primer design represents an upgrade in the stability of the blocking group and was developed specifically for use with high fidelity DNA polymerases that contain a 3’ exonuclease function. (**D**–DNA base complimentary to target, **M**–DNA base that is a mismatch to target, **r**–RNA base complimentary to target, **x**–C3 Spacer.

Removal of samples associated with false positive antibody titers will reduce the number of antibodies that need to be screened in downstream applications, thereby allowing for savings in reagent costs, screening time, and use of automation resources. In addition, accurate quantification of antibody titers will provide improved dose response data in downstream screening assays.

rh-PCR has been shown to reduce the formation of primer dimers and mis-primed PCR products, as well as to increase the accuracy of multiplex DNA amplification, and the recovery of low abundance targets [14]. Primers annealing to the FR1 and J-regions of the VL and VH chains were designed as rh-PCR Generation 1 (rh-PCR Gen-1) and Generation 2 (rh-PCR Gen-2) primers using commercially available ribonucleotides and blocking groups (Fig 2).

**Fig 3:**
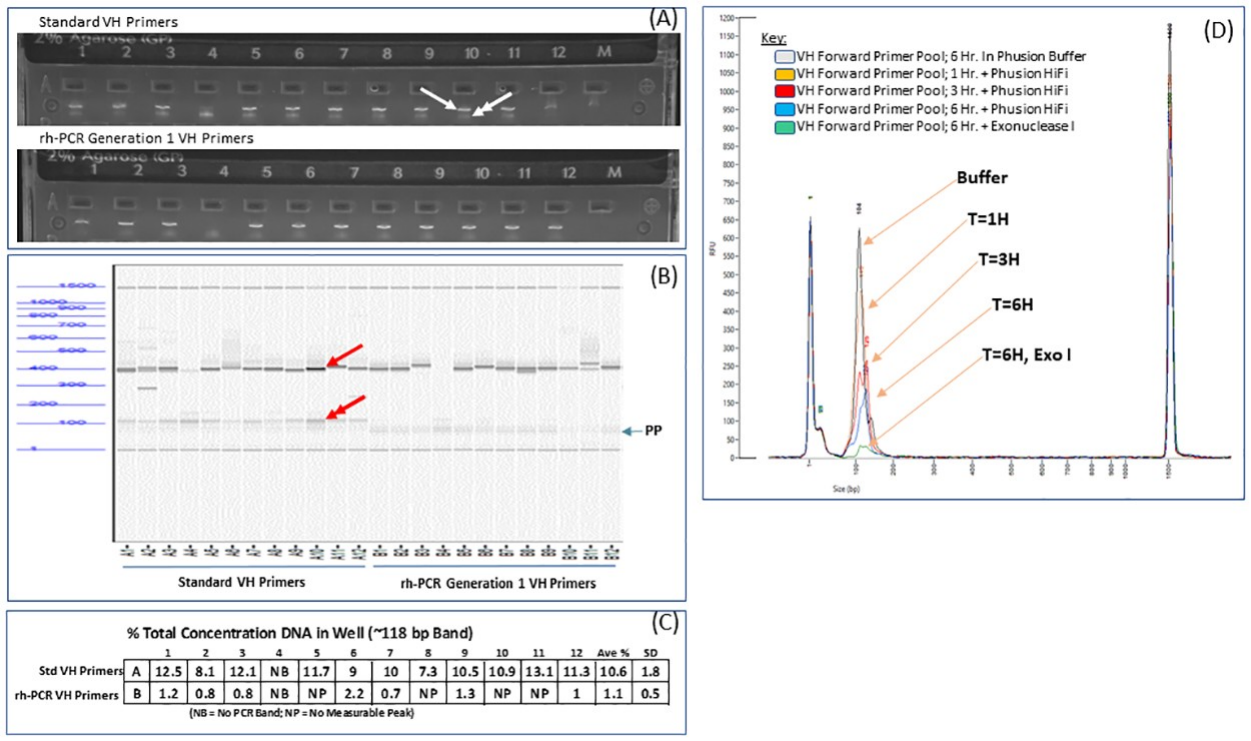
Analysis of standard and rh-PCR Gen-1 and primers for recovery of antibody variable domains. Amplification of VH regions using standard FR1 and J-regions primers or the rh-PCR Gen-1 variants as detected on an agarose gel (A) and by capillary electrophoresis (B). Full length VH fragment (arrowhead), small PCR product (double arrowhead) and unused Primer Pool (PP) are indicated. (C) quantification of truncated PCR products as a percentage of total DNA in each well following amplification of VH fragments using rh-PCR Gen-1 primers (D) Sensitivity of the rh-PCR Gen-1 primers to the 3’ exonuclease activity of the HiFi Phusion DNA polymerase and Exonuclease I.

Amplification of VH fragments by the rh-PCR Gen-1 primers was compared to that of the standard FR1 and J-region primers used in the nested PCR step using both agarose gel and capillary electrophoresis (Fig 3A and 3B). Visual inspection of the gel images and electropherogram plots suggested equivalent amounts of the full-length VH fragment and a reduction in small PCR product using the rhPCR-Gen1 primers. Quantification of the <150 bp PCR fragment (Fig 3C) demonstrated an almost 10-fold decrease in abundance of the small fragment using the rh-PCR Gen-1 primers (1.1%) relative to the standard primers (10.6%). The 3’exo-nuclease activity of some high-fidelity DNA polymerases has been implicated in the removal of the 3’ blocking group (rDDDDM-x) of the rh-PCR Gen1 primers (www.idtdna.com). A time course experiment in which the rh-PCR Gen1 forward primer pool (VH) was incubated with the HiFi Phusion enzyme used in the PCR #2 step (Fig 3D) was conducted. Changes in the size and shape of the primer peak with increasing incubation times confirms that the HiFi Phusion enzyme used in the Nested PCR can remove the 3’ blocking group at the end of the Gen-1 primers leading to degradation of the primers. Incubation of the rh-PCR Gen-1 primer pool with Exonuclease I, a single-strand DNA-specific enzyme with 3’ exonuclease activity resulted in almost complete degradation of the Gen-1 primer pool following six hours of incubation. Removal of the 3’ blocking group in the rh-PCR Gen-1 primers allows them to function as standard primers and may explain why the truncated PCR fragments observed were not completely eliminated.

**Fig 4:**
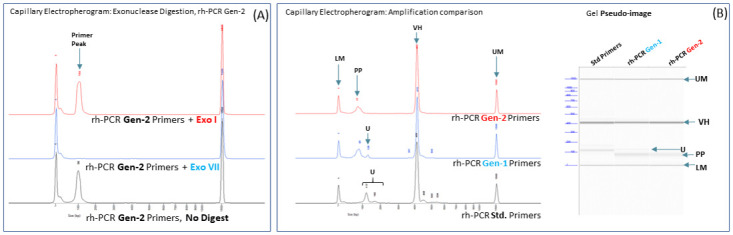
Characterization of rh-PCR Gen-2 primers. (A) Incubation of the VL rh-PCR Gen-2 primer pool with Exonuclease I or VII. (B) Amplification of VH fragments using standard FR1 and J-region PCR primers and the rh-PCR Gen-1 and rh-PCR Gen-2 derivatives. The electropherogram is on the left and a gel pseudo-image on the right. UM- CE Upper Marker, LM- CE Lower Marker, VH- variable region PCR product, PP- unused Primer Pool, U- Unwanted products.

To further reduce the presence of truncated PCR fragments to background levels, the stability of the 3’-blocking group of rh-PCR Gen2 primers was evaluated. In sharp contrast to the rh-PCR Gen-1 primers, incubation of the rh-PCR Gen-2 primers with Exonuclease I did not result in appreciable degradation of the primers (Fig 4A), suggesting greater stability of the 3’ blocking group (rDxxDM). As expected, the rh-PCR Gen-2 primers were digested from the unprotected 5’ terminus using the Exonuclease VII enzyme, a single-strand DNA nuclease with both 5’ and 3’ exonuclease activity.

Titration experiments with the RNase H2 enzyme confirmed that removal of the 3’ blocking group is absolutely required for PCR and identified 20 mU and 30 mU per reaction as the optimal enzyme concentrations for amplification of the VL and VH chains respectively using the rh-PCR Gen-2 primers (S2 Fig). Similar amounts of full-length VH fragments were obtained using the standard FR1 and J-region primers, or the rh-PCR Gen-1 or rh-PCR Gen-2 primer variants (Fig 4B). Analysis of the PCR products by capillary electrophoresis confirmed that rh-PCR Gen-1 primer pool dramatically reduced the amounts of unwanted truncated products relative to the standard primers, while the rh-PCR Gen-2 primers reduced the presence of truncated products to baseline (Fig 4B). Incubation of the rh-PCR Gen2 primer pool with Exonuclease VII identified the peak to the right of the Lower Marker as unused primer (Primer Peak) (Fig 4A). Given the greater stability of the 3’ blocking group on the rh-PCR Gen-2 primers and their ability to reduce the presence of unwanted truncated products to baseline levels, the utility of the rh-PCR Gen-2 primers to our antibody discovery process was tested in additional experiments.

### Amplification of VL/VH domains using rh-PCR Gen2 primers

**Fig 5:**
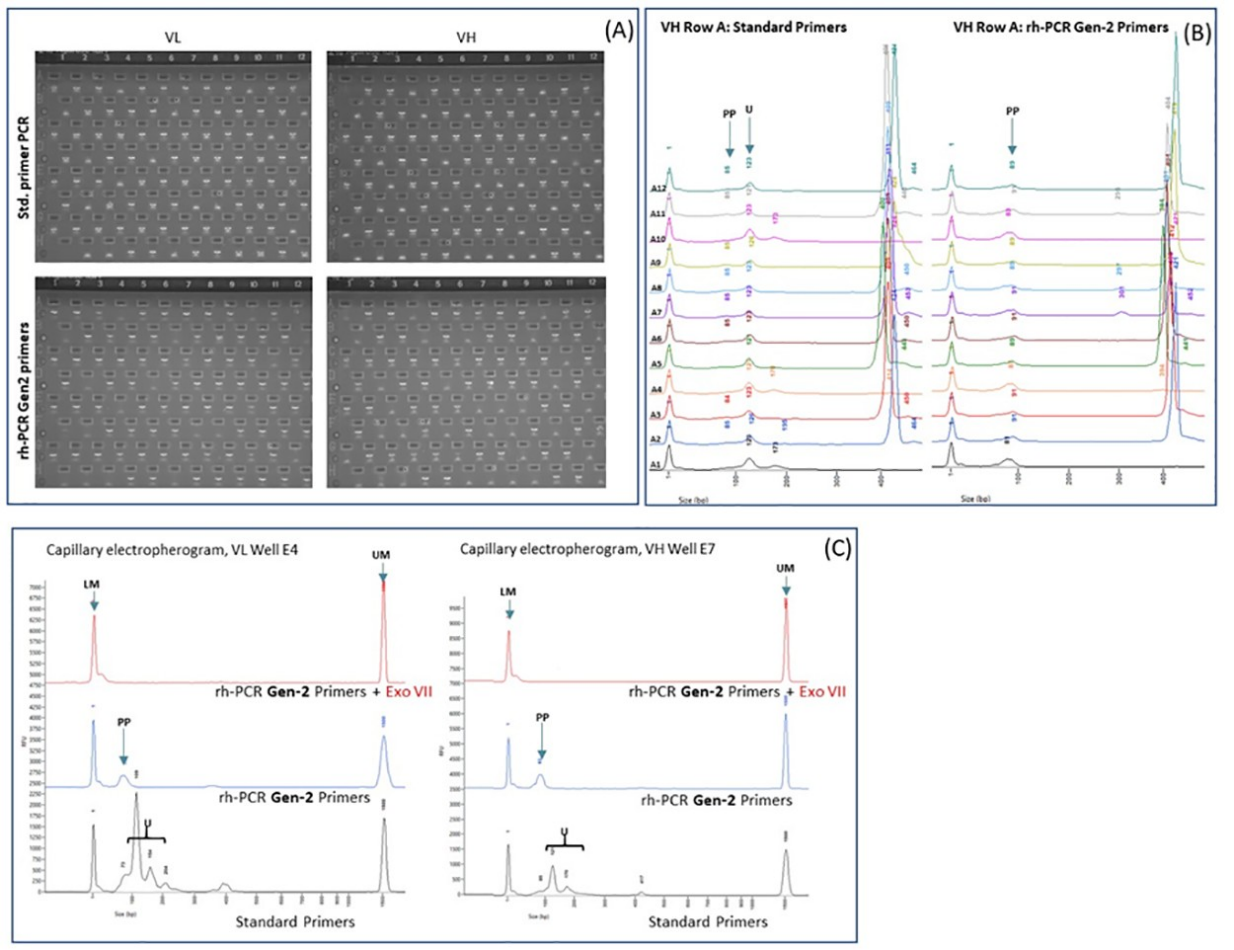
Characterization of VL and VH amplicons following amplification with rh-PCR Gen-2 primers. (A) Agarose gels of standard primers (top row) or rh-PCR Gen-2 primers (bottom row). Gels of the VL fragments are on the left and of the VH fragments on the right. (B) Electropherograms comparing a series of amplicons produced using standard primers (left) to those produced using rh-PCR Gen-2 primers (right). Truncated PCR products (U- Unwanted products) and unused primers (PP- Primer Pool) are denoted by arrowheads. (C) Comparison of wells lacking a full length VL (left) or VH (right) products amplified with either standard or rh-PCR Gen-2 primers. Truncated PCR products and unused primers are captured by a parenthesis and a single arrowhead, respectively. UM- CE Upper Marker, LM- CE Lower Marker, VH- variable region PCR product, PP- unused Primer Pool, U- Unwanted products.

Nested PCR amplicons obtained using the standard FR1 and J-region primers were compared to those using the rh-PCR Gen-2 primers across a series of VL and VH samples. PCR products were analyzed on agarose gels and by capillary electrophoresis (Fig 5A). The standard primers produced 79 VL and 60 VH PCR bands with 59 cognate VL/VH pairs. The rh-PCR Gen-2 primers produced 79 VL and 61 VH bands with 60 cognate VL/VH pairs. Analysis of a series of electropherograms revealed that the rh-PCR Gen-2 primers resulted in a single, full-length VH species with essentially no detectable truncated PCR products across a range of different antibody templates (Fig 5B and S3 Fig). In contrast, a small amount of the truncated PCR fragment was seen on the electropherograms when standard primers were used in the nested PCR, even in the presence of a full-length VH fragment. Treatment of the rh-PCR Gen-2 amplicon reactions with Exonuclease VII confirmed that the small peak running to the right of the 100 bp marker represents the unused primer pool (Fig 5C).

In cases where there was no recovery of full-length VL or VH amplicons (Fig 5C), PCR #2 with standard primers resulted in a significant number of unwanted products in both the VL and VH amplification reactions, while the rh-PCR Gen-2 primers had no detectable truncated products. Treatment of the rh-PCR Gen-2 amplicons with Exonuclease VII confirmed that the small peak running to the right of the lower marker (LM) represented unused primers.

### Use of rh-PCR Gen-2 primers allow for accurate identification of IgG+ wells

**Fig 6:**
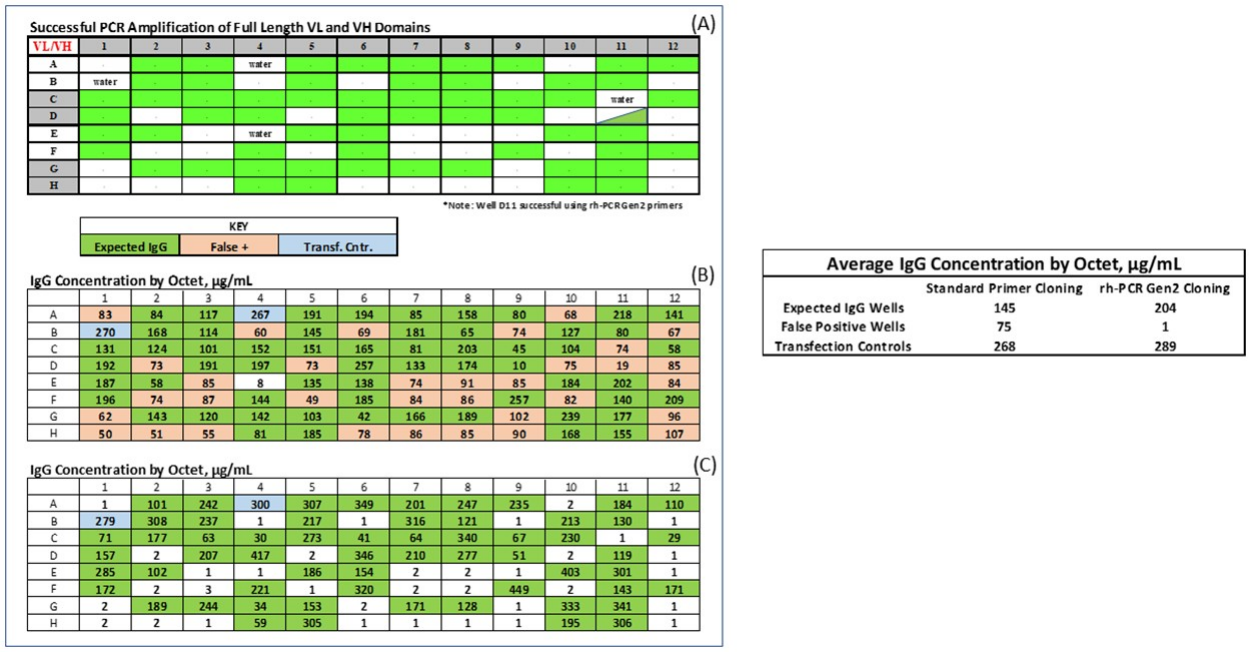
IgG titers following amplification of VL and VH regions using standard FR1 and J-region primers or their rh-PCR Gen-2 derivatives. Full-length VL and VH amplicon pairs using the standard primers are denoted by the green box (A). Antibody titers following VL and VH PCR using the standard primers (B), or the rh-PCR Gen-2 primers (C).

The VL and VH fragments amplified by either the standard FR1 and J-region primers or the rh-PCR Gen-2 variants (Fig 6A) were cloned into mammalian expression vectors. DNA was isolated from bulk *E*. *coli* transformants, the cognate VL/VH pairs mixed in the appropriate ratio and transfected into CHO cells in a 96-well microtiter plate. Antibody titers were determined using Octet biosensors six days post-transfection (Fig 6B and 6C). The average titers for the positive control plasmid were similar for the standard primer (269 μg/mL) and rh-PCR Gen-2 treatment plates (290 μg/mL). The average antibody titers for test samples containing plasmids with full-length VL and VH domains was 145 μg/mL for the standard primers and 204 μg/mL for the rh-PCR Gen-2 primers. Significantly, the average titers for wells containing plasmids missing at least one of the VL or VH fragments was 75 μg/mL using the standard primers and < 5.0 μg/mL using the rh-PCR Gen-2 primers. Under the conditions we run the Octet, antibody titers below 5 μg/mL are considered background. As observed in the initial experiments, in every well where standard primers were used to amplify the VL/VH products, antibody titers were > 5 μg/mL even when CHO cells were transfected with at least one truncated VL or VH domain. In direct contrast, only wells transfected with a full length VL and VH chain resulted in IgG titers > 5 μg/mL when rh-PCR Gen-2 primers were used in the nested PCR. A comparison of antibody titers across five plates from three different projects demonstrated an average increase of 56μg/mL in titers when rh-PCR Gen-2 primers were used as compared to the standard primers (Table 2).

**Table 2:** Antibody titers following amplification of VL/VH fragments using either standard or rh-PCR Gen-2 primers for the nested PCR.

Project	Plate	Std Primers	rh-PCR Primers	Ave. [IgG] Gain
**1**	**2**	**96**	**144**	**48**
**1**	**3**	**174**	**144**	**-30**
**1**	**4**	**177**	**216**	**39**
**2**	**17**	**212**	**377**	**165**
**3**	**3**	**145**	**204**	**59**
**Ave**	**5 Plates**	**161**	**217**	**56**

With two exceptions, the standard primers and rh-PCR Gen-2 primers returned the same wells with full-length VL/VH domains. In one case, (well 11D), the rh-PCR Gen-2 primers amplified a full-length VH fragment and the standard primers did not, while in the other in the case (well 6G) the reverse was true (Fig 6A).

### Identification of antigen positive binders

**Fig 7:**
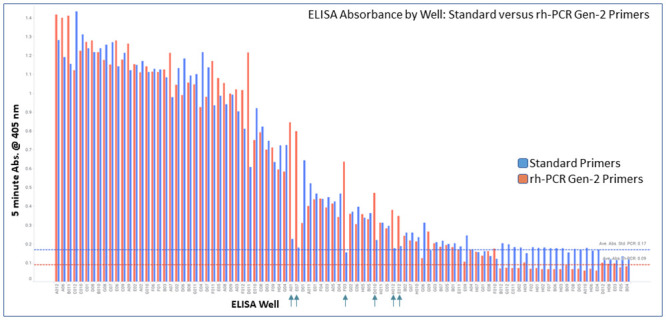
Ability of antibodies cloned from amplicons using standard primers or the rh-PCR Gen-2 variants to bind antigen in an ELISA format. Graphed by well in descending order of signal. Blue bars correspond to PCR #2 with standard primers, while orange bars correspond to PCR #2 with rh-PCR Gen-2 primers for the same samples. Blue and red dotted lines denote the average absorbance for unsuccessful PCR wells using the standard and rh-PCR Gen-2 primers, respectively. Newly identified antigen+ wells from rh-PCR Gen-2 supernatants are indicated by vertical arrows.

Antibodies derived from nested PCR with either the standard primers or the rh-PCR Gen-2 primers were tested for specific binding to the target protein directly immobilized on microtiter plates by ELISA. Antibodies were normalized to 5 μg/mL and then diluted 4-fold in a titration experiment. Antigen binding data at the highest antibody concentration is shown (Fig 7). ELISA signal intensities were similar across the primer sets. Of the 59 wells originally identified as having full-length VL and VH cognate pairs using the standard primers (Fig 1B), 52 wells were antigen positive using the standard primers, and 57 wells were antigen positive using the rh-PCR Gen-2 primers. A close examination of the electropherograms for the additional five antigen positive wells obtained with the rh-PCR Gen-2 primers identified a weakly amplified full-length VH fragment that could be “rescued” in the absence of interfering unwanted fragments (S4 Fig). The lack of truncated PCR products appeared to allow for greater sensitivity of the amplification reaction in the presence of rh-PCR primers. In general, non-antigen binding wells from amplifications using the standard primers had a higher background signal than those from rh-PCR primers.

### NGS of VH domains amplified using standard or rh-PCR Gen-2 primers

**Fig 8:**
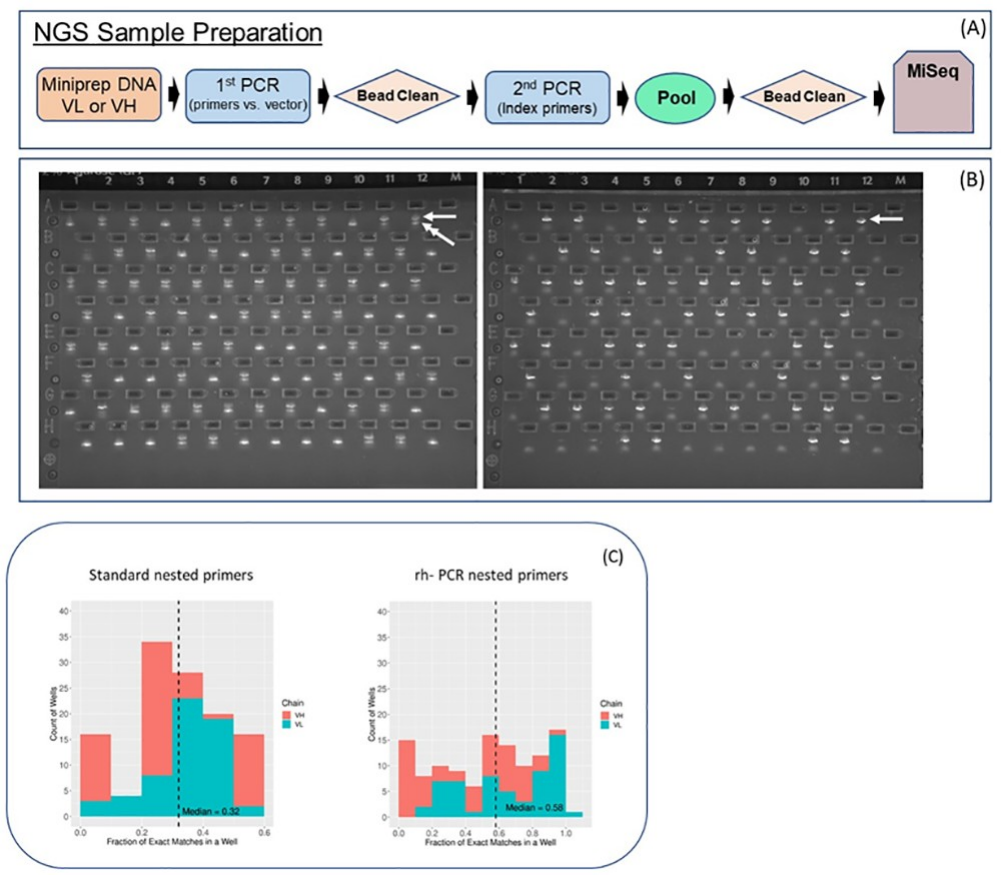
Next Generation Sequencing of amplicons derived from standard and rh-PCR Gen-2 primers. (A) schematic representation of the workflow. (B) Agarose gels of VH regions amplified (1^st^ PCR) from miniprep DNA cloned from either the standard nested primers (left) or the rh-PCR Gen 2 primers (right). Full length VH fragment (arrowhead), small PCR product (double arrowhead). (C) Fraction of reads for the major clonotype in each well matching the expected germline sequences in the first 21 nucleotides of framework 1. The distribution of sequences is compared using the standard nested PCR primers (left) or the rh-PCR primers (right) for both VL (turquoise) and VH (red) sequences.

As an alternative to Sanger sequencing of single *E*. *coli* colonies, NGS was used as a high-throughput means of obtaining sequence information on the antibody fragments amplified by either the standard or rh-PCR Gen-2 primers (Fig 8A). Antibody VL or VH fragments were amplified from the same bulk plasmid DNA used to transfect CHO cells using vector-specific primers hybridizing directly adjacent to the antibody variable regions. As expected, amplification of variable regions from bulk DNA having VH fragments amplified with standard FR1 and J-region primers contained full-length product, as well as the smaller truncated fragment. In contrast, VH fragments amplified with rh-PCR Gen-2 primers returned an apparent single fragment of the expected size (Fig 8B). After a bead clean-up step, a second PCR was used to bar-code each amplicon with a well-specific index tag of 10 nucleotides at both the 5’ and 3’ termini, as well as to add sequences required for Illumina sequencing. Analysis of the NGS reads from the plasmid DNA demonstrated that the total number of sequences obtained from the 59 wells was similar for both the VL and VH amplicon regardless of the primers used in the nested PCR (Table 3). However, a significant increase in the number of full-length antibody sequences was observed when the rh-PCR Gen2 primers were used in the PCR #2. In the case of the VL fragments the use of the rh-PCR Gen2 primers increased the average percentage of full-length antibody sequence reads as a fraction of total reads from 86.5% to 97.7%, while the percentage of wells in which all reads corresponded to full-length antibodies increased from 1.7% to 83.1%. In parallel, the rh-PCR Gen 2 primers increased the average percentage of full-length VH sequence reads from 83.9% to 98.4%. Significantly, in the case of the standard primers, none of the wells containing amplicons from the standard primers returned ≥ 95% full-length reads while in the case of the rh-PCR primers most wells (93.2%) returned entirely full-length antibody reads. Optimization of the number of full length sequences is important because it increases the efficiency of the sequencing run and allows for more samples to be processed.

**Table 3:** Next Generation Sequencing analysis of VL and VH regions following PCR #2 using standard or rh-PCR primers. Amplicons for NGS were prepared following cloning of PCR fragments into mammalian expression vectors as outlined in Fig 8A. The average number of full-length reads was determined based on the percentage of full length reads for each of the 59 wells tested.

	VL Chain		VH Chain	
Reads (n = 59 wells)	Standard primers	rh-PCR primers	Standard primers	rh-PCR primers
**Total number of MiSeq reads**	**343,399**	**355,141**	**336,632**	**336,262**
**Average full-length Ab reads (%)**	**86.5**	**97.7**	**83.9**	**98.4**
**Median full-length Ab reads (%)**	**90**	**100**	**86**	**100**
**Range of full-length Ab reads (%)**	**42–100**	**54–100**	**52–93**	**57–100**
**Wells ≥ 90% full length reads (%)**	**50.8**	**91.5**	**16.9**	**96.6**
**Wells ≥ 95% full length reads (%)**	**20.3**	**91.5**	**0**	**94.9**
**Wells = 100% full length reads (%)**	**1.7**	**83.1**	**0**	**93.2**

Next, the standard primers were compared to the rh-PCR primers with regards to the number of recovered VH and VL genes having the correct germline sequence in the primer annealing region of FR1 (first 21 nucleotides). In this analysis, the VL or VH gene with the largest number of reads in each well was grouped with sequences having the same VL-CDR3 or VH-CDR3 sequences (closely related clonotypes), and the number of reads having the correct germline sequences determined as a proportion of the total number of reads for that clonotype in a particular well (Fig 8C). When VL/VH fragments were amplified using the standard PCR, a median of 32% of the dominant clonotypes matched the expected germline sequences in the first 21 bases of the FR1 hybridized by the primers. In sharp contrast, the rh-PCR primers resulted in 58% of all sequences having the correct germline sequences. Concomitantly there was a significant increase in the fraction of wells (>0.6) having sequences matching the native germline sequences. Together these results confirm the increased specificity of the rh-PCR primers. The VL rh-PCR primers retained the expected germline sequences to a greater extent than the VH primers. Interestingly, in some cases, none of the sequences at the 5’ terminus of the FR1 region were a perfect match to the expected germline VL or VH sequences.

## Discussion

This research demonstrates the advantages of using rh-PCR primers in the high-throughput cloning of antibody variable regions from single B-cells. We establish that rh-PCR primers in the amplification reactions results in reduction of primer dimers to below detectable levels. This in turn, eliminates false positive antibody titers using ProG sensors on the Octet in cases where primer dimer fragments are cloned into mammalian expression vectors in-frame with the upstream signal sequence and downstream antibody constant domain sequences. The greater specificity and robustness of rh-PCR relative to standard primers results in increased antibody titers and a higher number of antigen-specific binders. Finally, we demonstrate that NGS of rh-PCR amplicon pools results in a higher number of full-length VL and VH sequencing reads and a greater proportion of sequences with correct germline sequences in the primer annealing region. This high-throughput sequencing approach provides an efficient alternative to Sanger sequencing of single *E*. *coli* colonies.

Animal species used in most antibody discovery platforms contain multiple germline VL and VH sequences. Consequently, multiple primers annealing to the signal peptide, FR1 and J-region antibody sequences are needed to recover as many antigen-specific genes from the antigen-experienced Ig-repertoire as possible. Primer design is constrained by the need to recover full-length, functional antibody sequences. Design is further limited by the fact that each primer must contain at its 5’-termini sequences that overlap the vector and allow for Gibson assembly. Finally, hairpin structures caused by nucleotide complementarity within the primer must be avoided, particularly at the 3’ terminus. In this report, full-length VL and VH sequences were recovered from 64% of the input B-cells. The efficiency of VL and VH gene recovery is highly dependent on the antigen used for the immunization, the robustness of the immune response, the immunization protocols used, the subtypes of B-cells isolated, and the isolation methods employed. The same primers outlined in this study have been used successfully on numerous occasions to isolate full length antibody genes from 80–90% of isolated B-cells, suggesting an overall robust primer design. The example provided in this study highlights the advantage of rh-PCR.

When large numbers of antibodies must be processed, timeline requirements and the cost of each Sanger sequencing reaction precludes the isolation and sequencing of individual *E*. *coli* colonies from each cloning reaction. In our protocols, Gibson assembly is used to clone the antibody variable domains into their respective expression vectors. Because plasmids are not purified from single *E*. *coli* colonies, it is important that full length VL and VH PCR fragments are cloned into expression vectors with high efficiency in order to maximize antibody titers as required for downstream assays. To this end, carefully optimized PCR conditions, insert-vector overlap sequences, and template: primer ratios have shown to be critical for cloning success. Efficient cloning of the VH domains was confirmed by the observation that >90% of the single colony sequences that were obtained as part of the optimization process were cloned correctly.

Multiplex PCR using a series of forward and reverse primers is challenging because all the primers must function under the same PCR conditions, formation of primer dimers should be minimized, primers must be economical for large scale purchase, and PCR conditions must be robust, reproducible and amenable to high-throughput protocols [40]. Given that that our high-throughput cloning protocols preclude the isolation of sequence-confirmed clones from single *E*. *coli* colonies, the elimination of primer dimers is particularly important to eliminate wells with a false-positive IgG titer reading. Numerous strategies to minimize primer dimer formation have been proposed including, modification of PCR conditions, addition of adjuvants, and fine-tuning of primer-template ratio’s [41, 42]. In addition, complex primer designs using 5’ “Tailed sequences” [43], or 3’ photo-labile protective groups have also been reported [44]. This research demonstrates a robust approach to eliminating primer dimer formation in the context of VL and VH sequence recovery from single B-cells and subsequent cloning of the fragments into mammalian expression vectors. The cost of rh-PCR primers is approximately 10-fold greater than that of standard primers (desalted) and addition of RNase H2 enzyme adds $0.07 to each reaction. However, these added costs are off-set by elimination of false positive antibody wells from downstream workflows. The time (employee and automation equipment) and cost savings achieved by the elimination of IgG false positive wells from the workflow will depend on the screening assays employed in the discovery project. Cell assays can be very expensive in terms assay reagents and cell line propagation, while the cost of ELISA’s are driven by the time and effort spent expressing, purify and QC’ing of the recombinant proteins. High throughput screening of antibodies typically involves 10- or 8- point concentration titrations against homologous targets from multiple species (human and mouse and cynomolgus monkey), further multiplying the number of plates that must be screened.

Antibody sequence information is a prerequisite for protein engineering efforts designed to improve the biophysical characteristics of an antibody, eliminate post-translational modification liabilities, and reduce potential for immunogenicity. Furthermore, sequence information is critical for the development of *in silico* models and understanding of analytical assays designed to improve manufacturing, dosing regimens and effectiveness of biotherapeutic antibodies. Sanger sequencing of single *E*. *coli* colonies following transformation of cloning reactions has traditionally been used to obtain antibody sequences for internal databases. However, performing Sanger sequencing and subsequent analysis of multiple colonies from 1000s of cloning reactions is labor intensive, time-consuming, and an expensive endeavor. Here we employ a well-specific index sequence to tag the VL and VH amplicons in each well and use an NGS approach to obtain sequence information from the VL and VH domains of each B-cell. The estimates derived from this work suggest that this high-throughput approach provides sequence information in less than half the time required for the Sanger approach and at approximately a third of the cost (S1 Table). In conjunction with data from analytical analysis and animal studies, facile and economical antibody sequencing from our discovery platforms will allow for the development of *in silico* tools for antibody property predictions on more molecules, which will enable productive binning strategies.

In this study we confirm the susceptibility of the rh-PCR GEN1 primers to 3’ cleavage of the blocking group by HiFi Phusion polymerase. The difference in exonuclease susceptibility between the rh-PCR GEN1 and rh-PCR GEN2 primer is related to the location of the C3 spacer, as well as the number of spacers in the primers (personal communication). In the rh-PCR GEN2 primers, the C3 spacers are back to back and much less recognizable by the 3’exonuclease than a single C3 spacer, as is the case of the rh-PCR GEN1 primers.

RNase H2 primers have been shown to increase the specificity of PCR with respect to off-target amplification. The efficiency of cleavage by the RNase H2 enzyme is ribonucleotide-dependent, with uracil being the least preferred residue (www.idtdna.com). In addition, the position of the mismatched base relative to the ribonucleotide has a significant effect on the efficiency of cleavage. Mismatches more than 3 nucleotides upstream of the ribonucleotide, or one nucleotide downstream (“-3, +1”) have been shown to have a minimal effect on RNase H2-mediated cleavage of the scissile bond [14]. In this study, this increased specificity of the rh-PCR was confirmed by demonstrating a significant increase in the number of clones whose sequences match the expected germline sequences in the FR1 region hybridized by the primers used in the nested PCR amplification. Although there are many germline sequences in the mouse, rabbit, and human immune repertoires, there are redundancies at the nucleotide level in the first 21 bases of the FR1 germline sequences. To reduce the number of primers in the forward pool, some mismatches between the primer and FR1 sequences, particularly towards the 5’ terminus were tolerated under the PCR conditions used. Consequently, not all VL/VH amplicons had a perfect match with their expected germline sequence. In most cases, the absence of any VL/VH amplicons with a perfect match to their expected germline sequence was due to the absence of a primer specific for that germline sequence. Examples where the correct primer was present in the reaction mix, but the expected germline sequences were either not observed, or present in low abundance will be particularly informative in terms of future primer design. Efforts are underway to improve the fidelity of amplification with regards to maintaining germline sequences through novel primer designs and modifications in PCR conditions. Nevertheless, the desire to finely tune the specificity of the amplification reaction, must be balanced against the need to optimize recovery of VL/VH fragments from large numbers of B-cells in a robust manner. This is of particular concern given the stochastic nature of PCR in cases where starting template concentrations are low [45–47].

In this study we apply rh-PCR to our antibody discovery platform and demonstrate its ability to eliminate the formation of primer dimers and increase recovery of templates present at low starting concentrations. rh-PCR is robust and easily incorporated into most commonly used PCR protocols. Using rh-PCR, we have successfully increased the number of target-specific antibodies recovered in our single B-cell discovery platform across more than 50 immunizations. The improved specificity of the rh-PCR primers is reflected in a more homogenous pool of DNA for downstream cloning and expression. We have taken advantage of the cloning of high-quality PCR products generated by rh-PCR to apply an NGS approach to sequence the cognate VL and VH antibody pairs from 1000s of individual B-cells efficiently and economically. We believe that the ability of rh-PCR to eliminate primer dimer formation, and increase the specificity fragment amplification, as well as its ease of use will be beneficial in other challenging multiplex PCR applications.

## Supporting information

S1 Fig Representative sequence data from a single clone containing a truncated VH PCR product. The 54 base pair insertion is in frame with the leader sequence and the constant region and expresses a shorter than full length heavy chain.
(TIF)

S2 Fig Titration of RNase H2 used in rh-PCR reactions. Each row of PCR reactions are derived from master mixes containing differing amounts of RNase H2 enzyme, from 0 to 100 mU per reaction. The required amount of RNase H2 needed for successful amplification, indicated by the arrow, was determined by comparing the number of observed bands at each test condition to that row when amplified with the maximum RNase H2 (100 mU). Gels not shown.
(TIF)

S3 Fig Characterization of VL amplicons following amplification with rh-PCR Gen-2 primers. Electropherograms comparing a series of amplicons produced using standard primers (left) to those produced using rh-PCR Gen-2 primers (right). Truncated PCR products (U- Unwanted products) and unused primers (PP- Primer Pool) are denoted by arrowheads.
(TIF)

S4 Fig Electropherograms of two PCR #2 amplifications. A poorly amplified variable region, right panel, and for comparison in overlay with a typical amplification product, left panel. The poorly amplified variable region was successfully cloned and expressed. Unused primers (PP- Primer Pool), lower marker (LM) and upper marker (UM) are denoted by arrowheads.
(TIF)

S1 Table DNA sequencing strategies for newly discovered antibodies. Sanger sequencing of single *E*. *coli* colonies as compared to a high throughput Next Gen Sequencing approach are compared for a typical discovery campaign.
(TIF)

S1 Raw images (PDF)
