# Supplementary figures and images for: RNase H-dependent PCR enables highly specific amplification of antibody variable domains from single B-cells
Source: PLoS One. 2020 Nov 5;15(11):e0241803. doi: 10.1371/journal.pone.0241803 (PMC7643965; doi:10.1371/journal.pone.0241803)

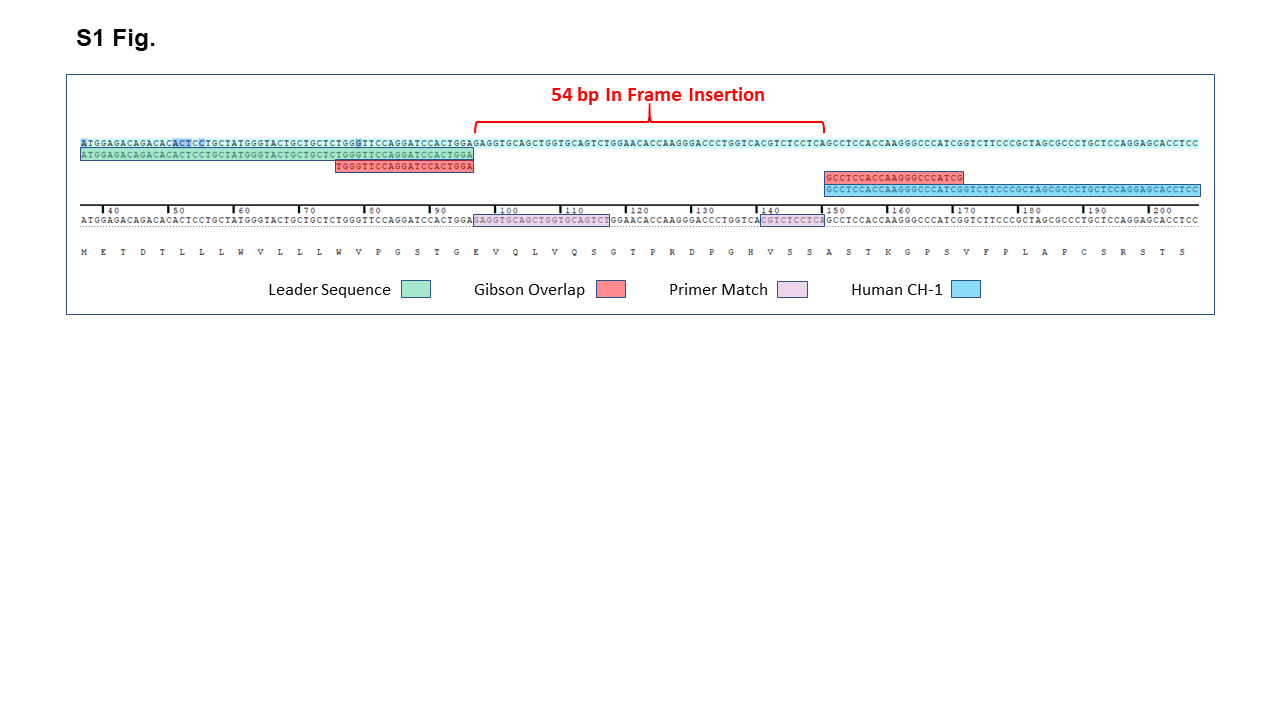

Supplement: S1 Fig — The 54 base pair insertion is in frame with the leader sequence and the constant region and expresses a shorter than full length heavy chain. (TIF) [file pone.0241803.s001.tif]

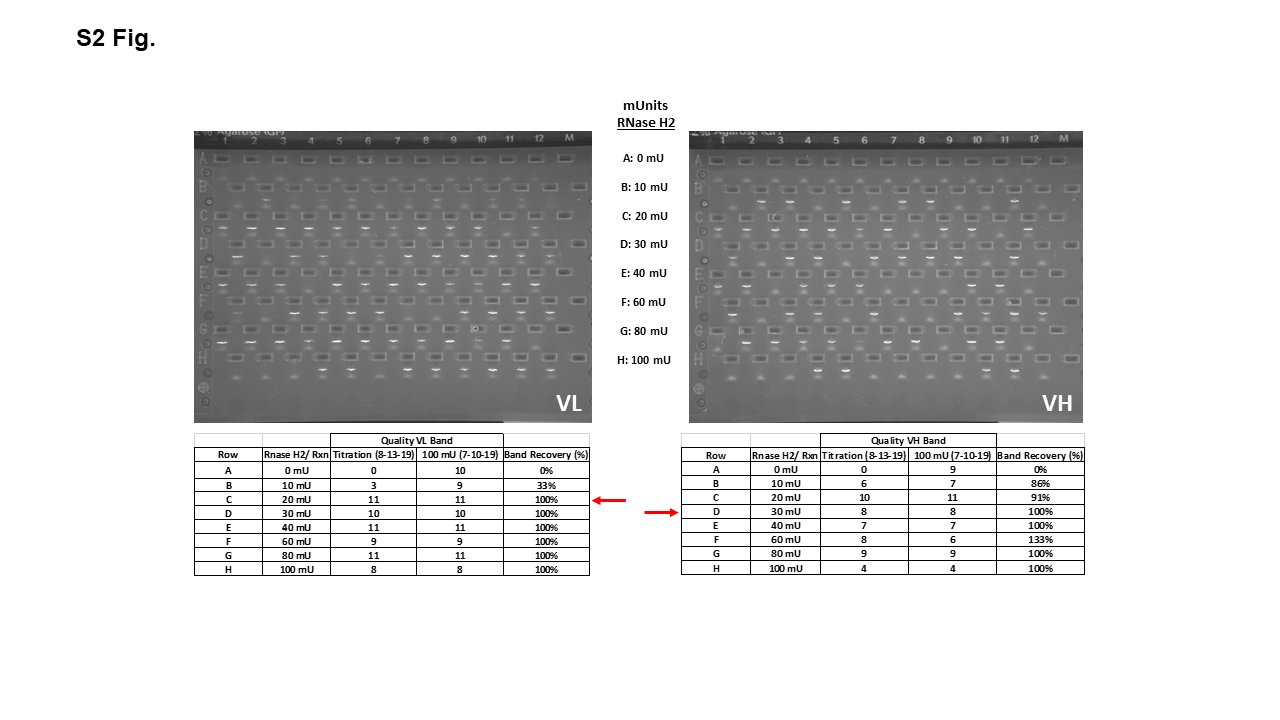

Supplement: S2 Fig — Each row of PCR reactions are derived from master mixes containing differing amounts of RNase H2 enzyme, from 0 to 100 mU per reaction. The required amount of RNase H2 needed for successful amplification, indicated by the arrow, was determined by comparing the number of observed bands at each test condition to that row when amplified with the maximum RNase H2 (100 mU). Gels not shown. (TIF) [file pone.0241803.s002.tif]

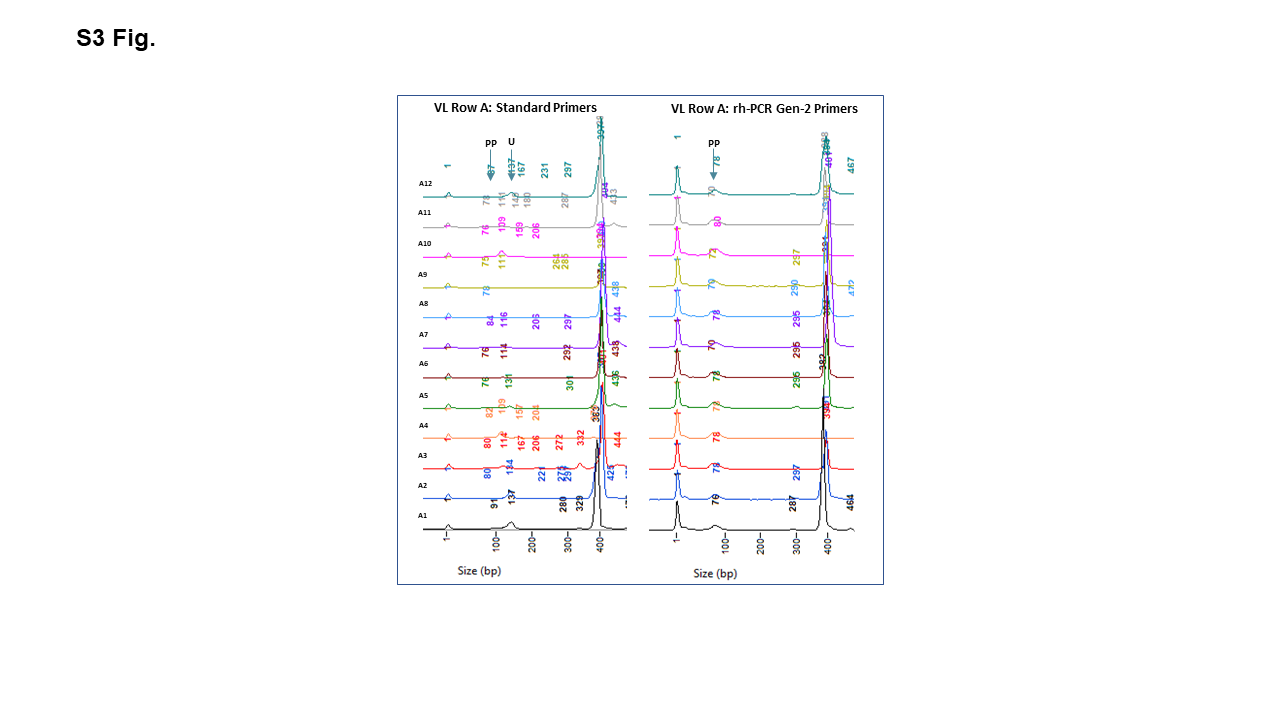

Supplement: S3 Fig — Electropherograms comparing a series of amplicons produced using standard primers (left) to those produced using rh-PCR Gen-2 primers (right). Truncated PCR products (U- Unwanted products) and unused primers (PP- Primer Pool) are denoted by arrowheads. (TIF) [file pone.0241803.s003.tif]

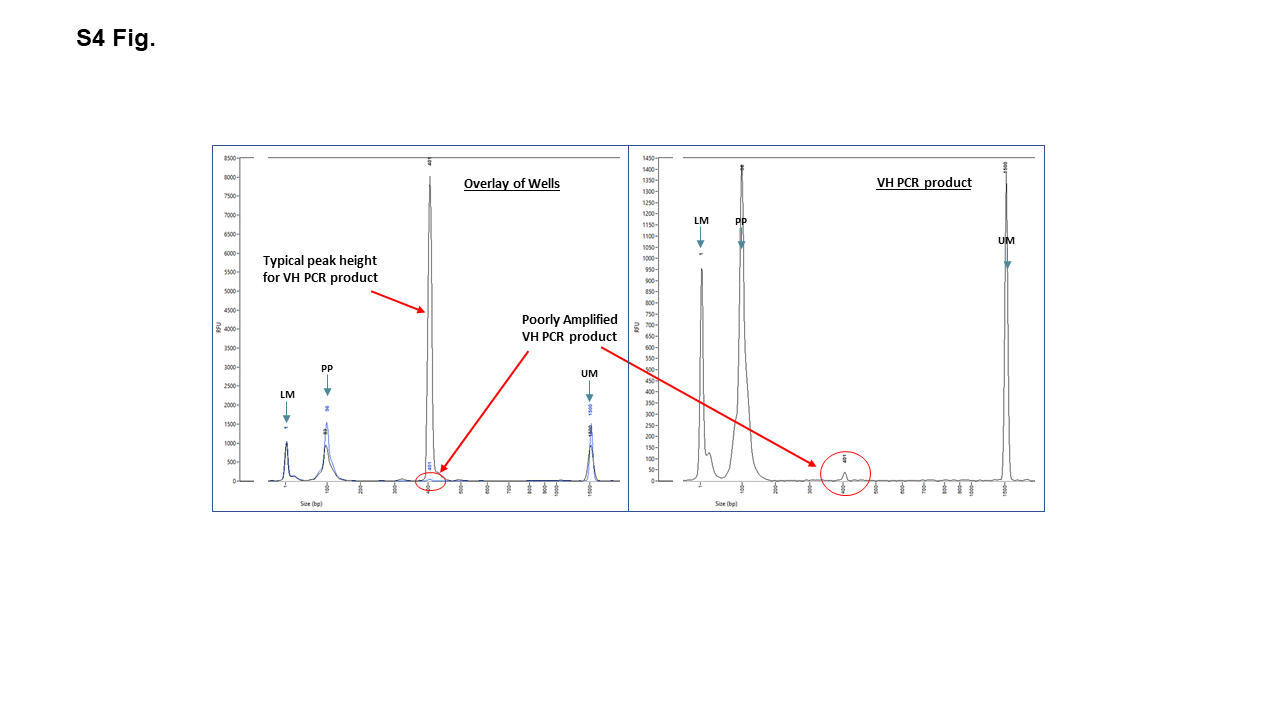

Supplement: S4 Fig — A poorly amplified variable region, right panel, and for comparison in overlay with a typical amplification product, left panel. The poorly amplified variable region was successfully cloned and expressed. Unused primers (PP- Primer Pool), lower marker (LM) and upper marker (UM) are denoted by arrowheads. (TIF) [file pone.0241803.s004.tif]

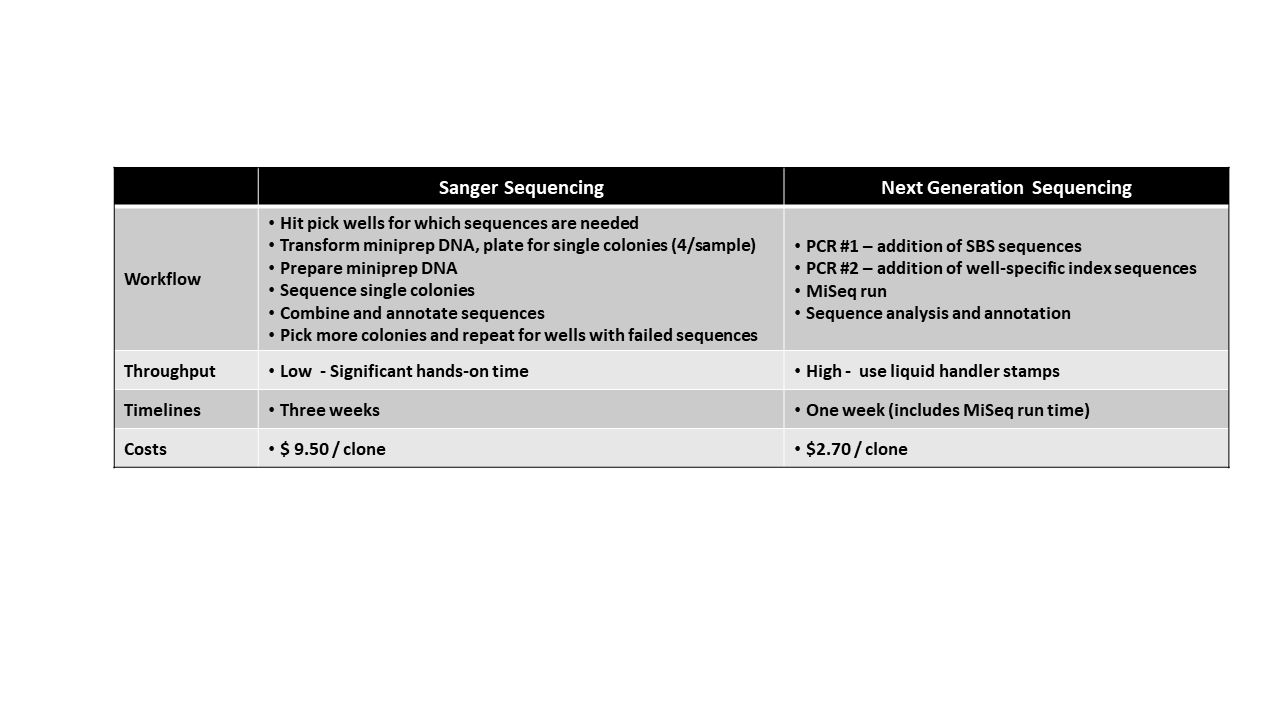

Supplement: S1 Table — Sanger sequencing of single E. coli colonies as compared to a high throughput Next Gen Sequencing approach are compared for a typical discovery campaign. (TIF) [file pone.0241803.s005.tif]
