# Supplementary material for: RNase H-dependent PCR enables highly specific amplification of antibody variable domains from single B-cells
Source: PLoS One. 2020 Nov 5;15(11):e0241803. doi: 10.1371/journal.pone.0241803 (PMC7643965; doi:10.1371/journal.pone.0241803)

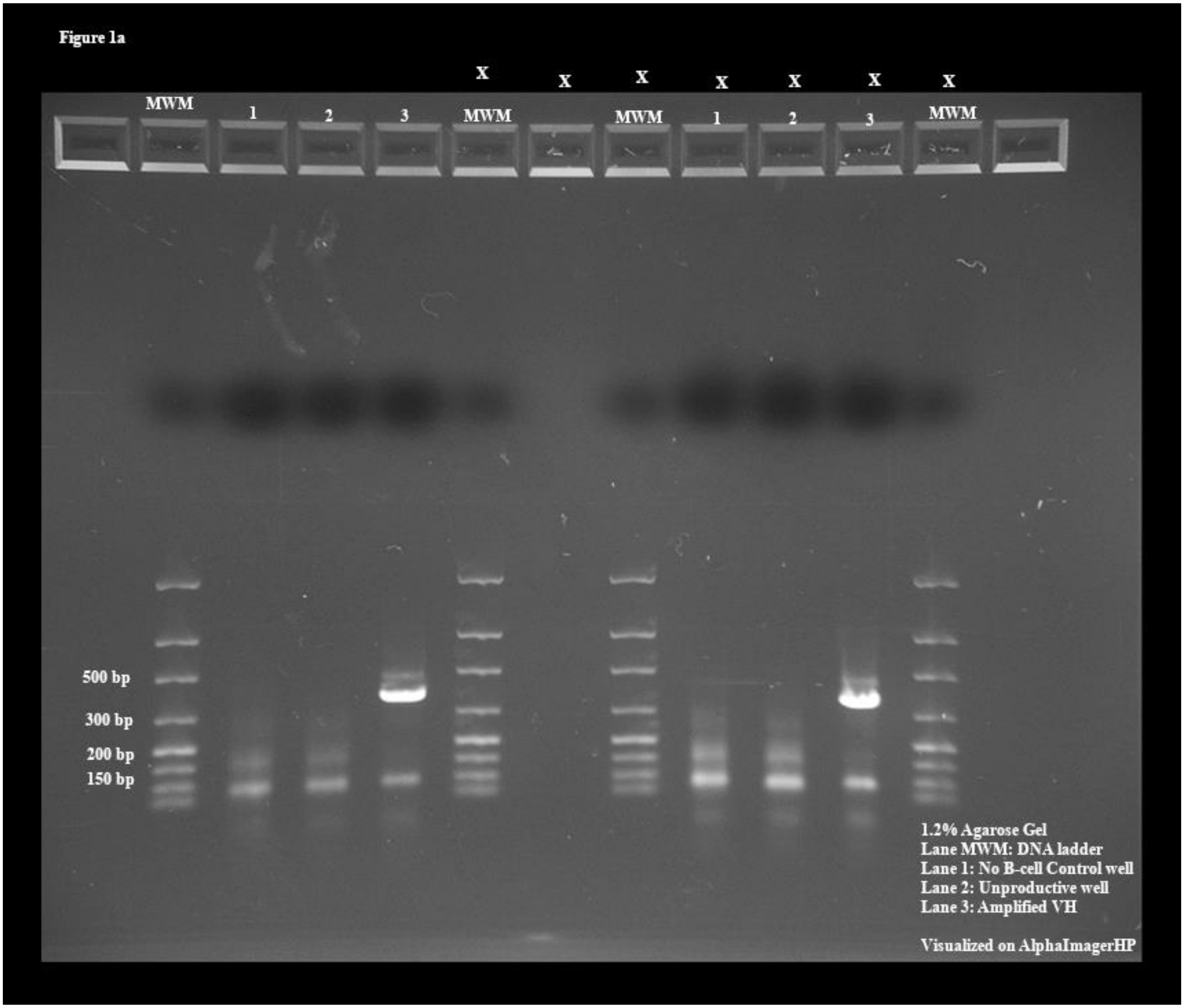
Figure 1a
X
X
X
X
X
X
X
MWM
1
2
3
MWM
MWM
1
2
3
MWM
500 bp
300 bp
200 bp
150 bp
1.2% Agarose Gel
Lane MWM: DNA ladder
Lane 1: No B-cell Control well
Lane 2: Unproductive well
Lane 3: Amplified VH
Visualized on AlphaImagerHP

FIGURE 1b

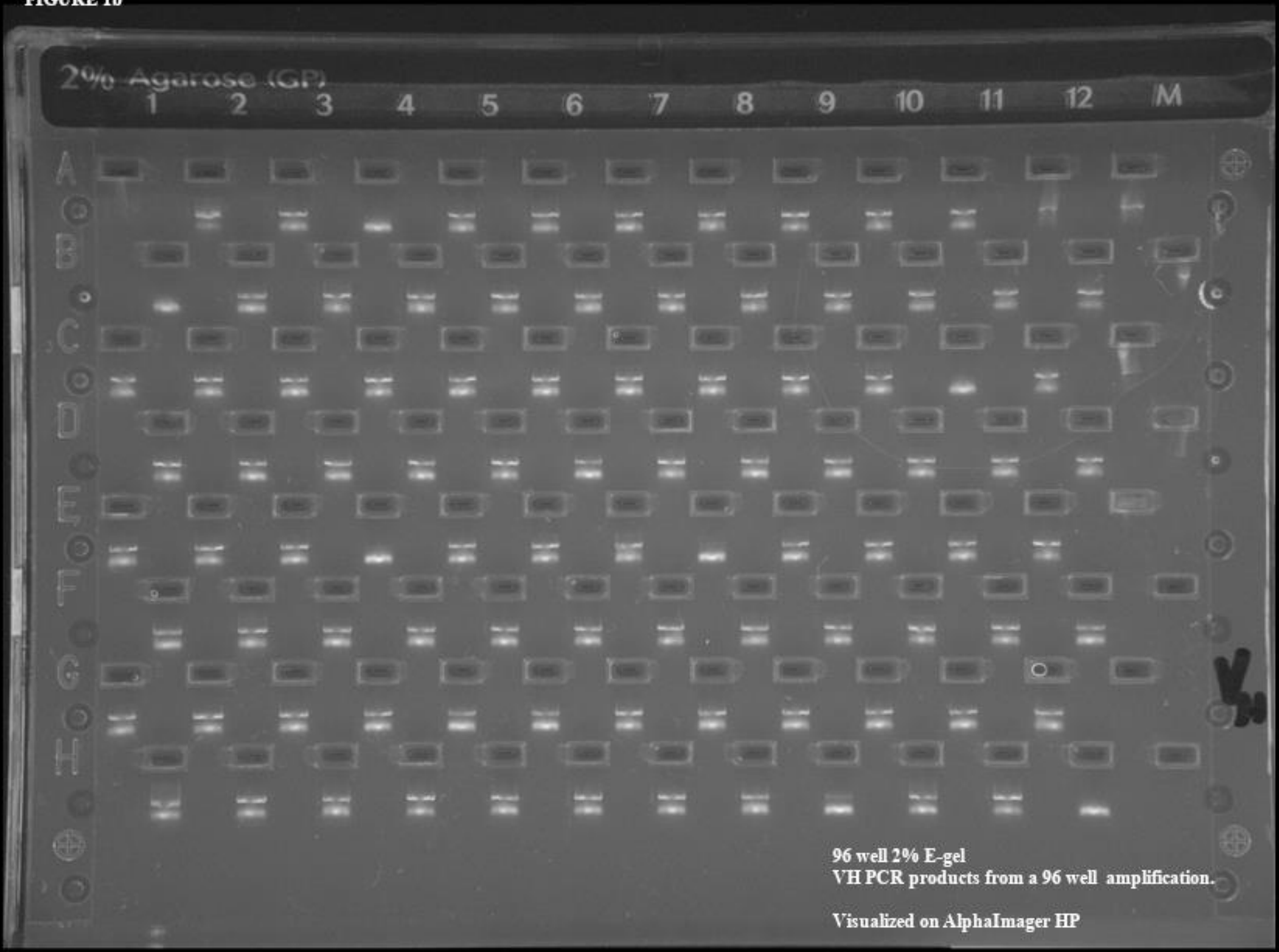
2% Agarose (GP)
1 2 3 4 5 6 7 8 9 10 11 12 M
A B C D E F G H
96 well 2% E-gel
VH PCR products from a 96 well amplification.
Visualized on AlphaImager HP

**FIGURE 1e:**

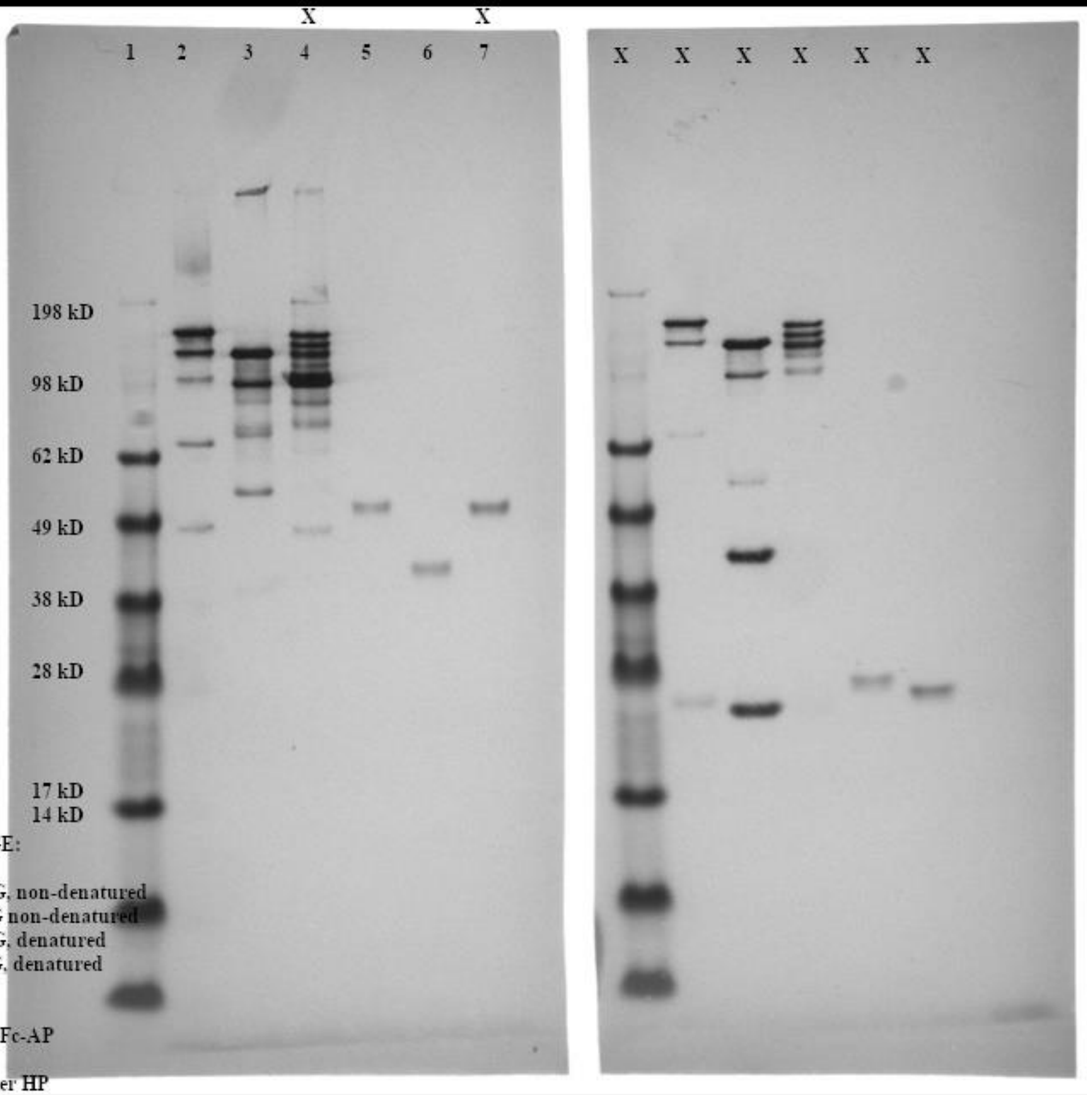


4-12% BIS-TRIS PAGE:
Lane 1: MWM
Lane 2- Full length IgG, non-denatured
Lane 3- Truncated IgG non-denatured
Lane 5- Full length IgG, denatured
Lane 6- Truncated IgG, denatured

Western detection:
Goat anti-human IgG-Fc-AP

Imaged on AlphaImager HP

FIGURE 3:

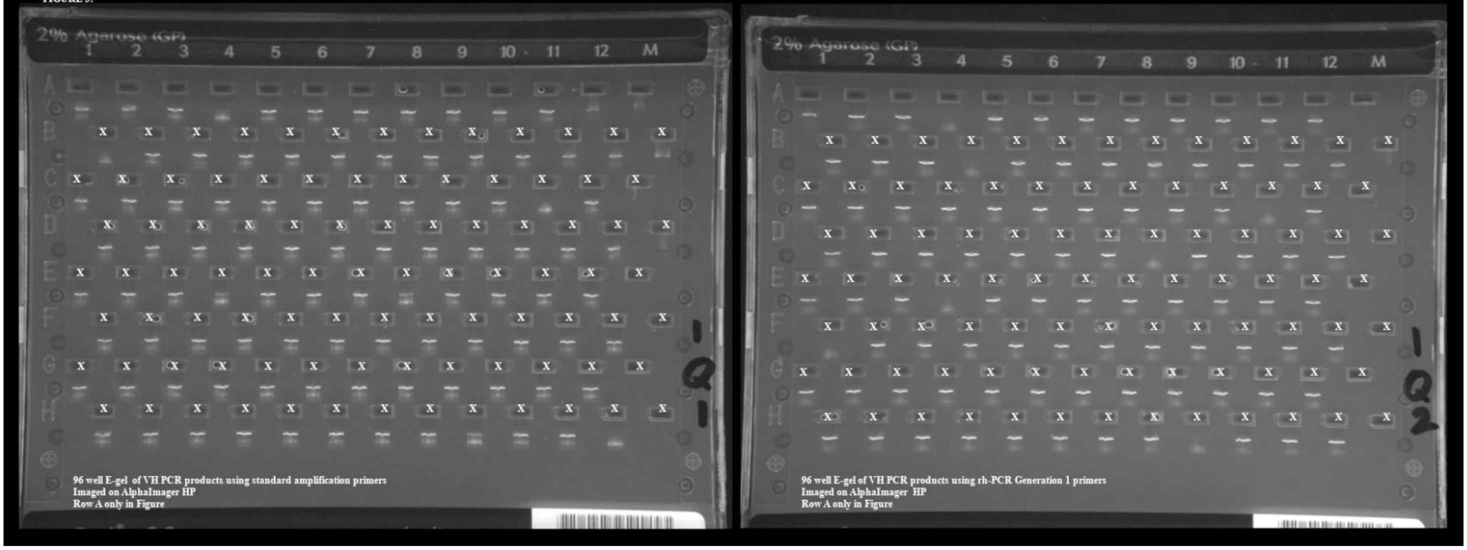
2% Agarose (GP)
1 2 3 4 5 6 7 8 9 10 11 12 M
A B C D E F G H
96 well E-gel of VH PCR products using standard amplification primers
Imaged on AlphaImager HP
Row A only in Figure
2% Agarose (GP)
1 2 3 4 5 6 7 8 9 10 11 12 M
A B C D E F G H
96 well E-gel of VH PCR products using rh-PCR Generation 1 primers
Imaged on AlphaImager HP
Row A only in Figure

FIGURE 5a

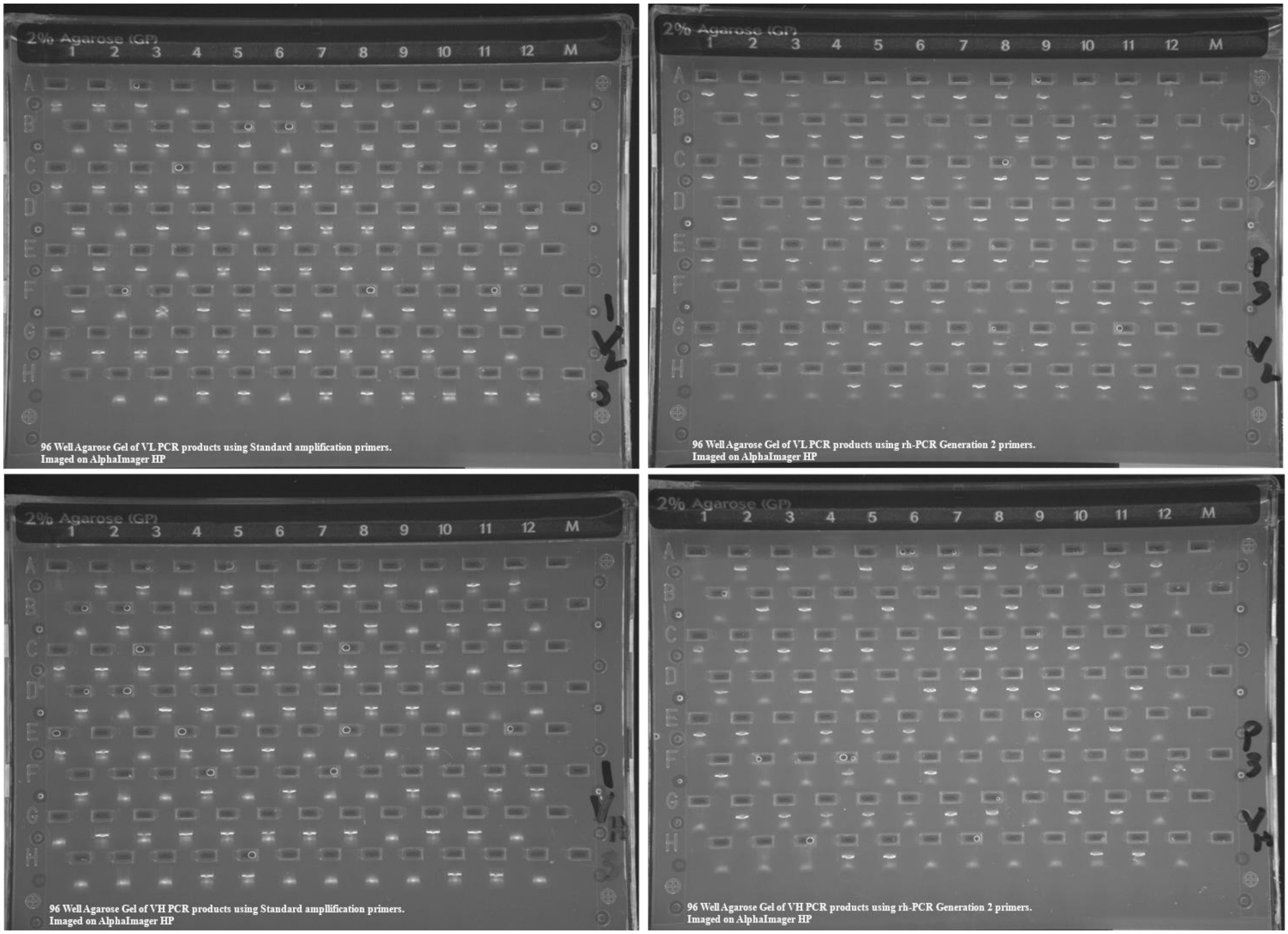


96 Well Agarose Gel of VL PCR products using Standard amplification primers.
Imaged on AlphaImager HP

96 Well Agarose Gel of VL PCR products using rh-PCR Generation 2 primers.
Imaged on AlphaImager HP

96 Well Agarose Gel of VH PCR products using Standard ampllification primers.
Imaged on AlphaImager HP

96 Well Agarose Gel of VH PCR products using rh-PCR Generation 2 primers.
Imaged on AlphaImager HP

FIGURE 8b

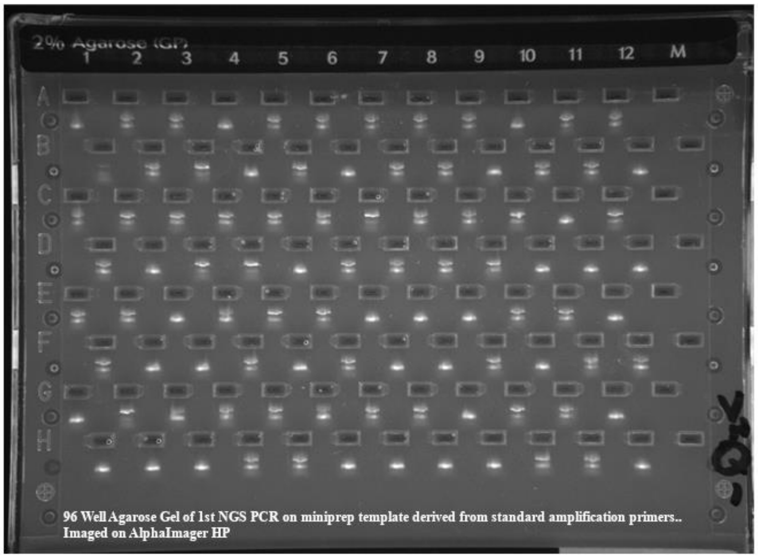


96 Well Agarose Gel of 1st NGS PCR on miniprep template derived from standard amplification primers..
Imaged on AlphaImager HP

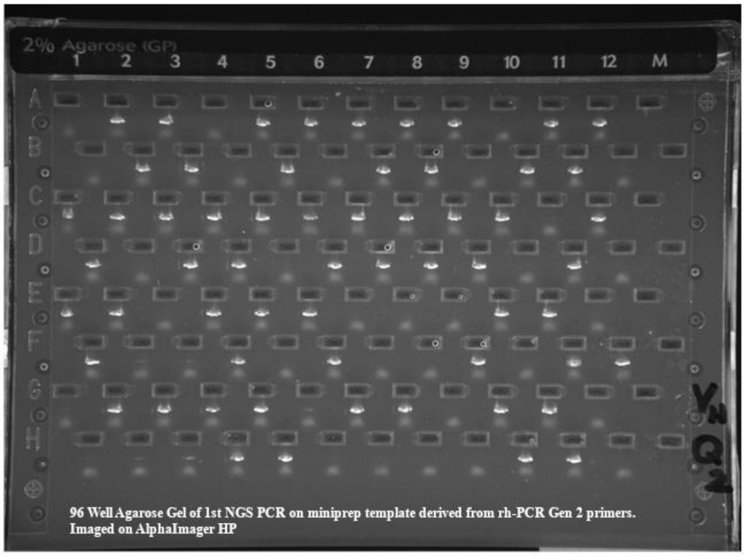


96 Well Agarose Gel of 1st NGS PCR on miniprep template derived from rh-PCR Gen 2 primers.
Imaged on AlphaImager HP

SUPPLEMENTAL FIGURE 2

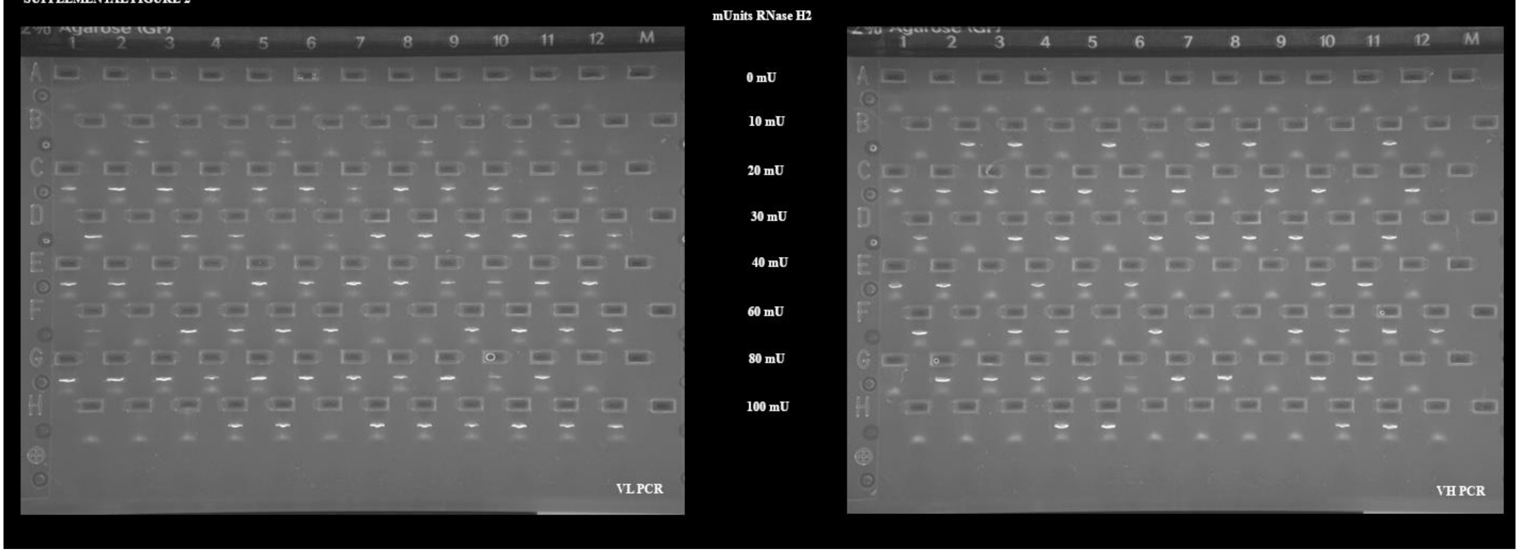

Supplement: S1 Raw images — (PDF) [file pone.0241803.s006.pdf]
